# A Near-Infrared Luminescent Cr(III) *N*-Heterocyclic Carbene Complex

**DOI:** 10.1021/acs.inorgchem.4c01270

**Published:** 2024-05-02

**Authors:** Robert W. Jones, Rory A. Cowin, Iona I. Ivalo, Dimitri Chekulaev, Thomas M. Roseveare, Craig R. Rice, Julia. A. Weinstein, Paul I. P. Elliott, Paul A. Scattergood

**Affiliations:** ‡ Department of Chemistry, University of Huddersfield, Queensgate, Huddersfield HD1 3DH, U.K.; § Department of Chemistry, University of Sheffield, Brook Hill, Sheffield S3 7HF, U.K.

## Abstract

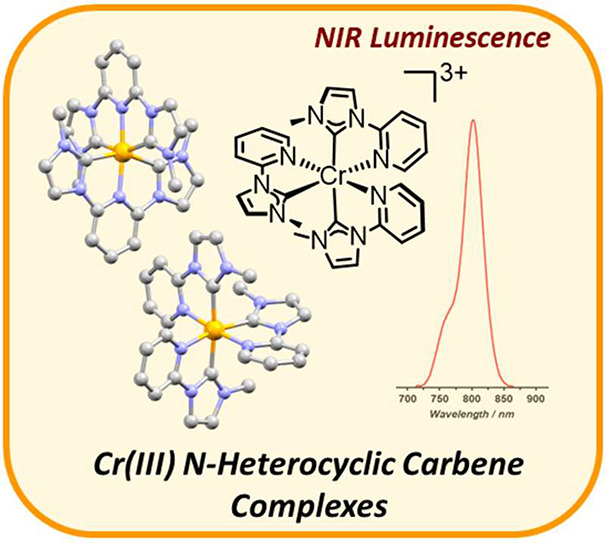

Photoluminescent coordination complexes of Cr(III) are of interest as near-infrared spin-flip emitters. Here, we explore the preparation, electrochemistry, and photophysical properties of the first two examples of homoleptic *N*-heterocyclic carbene complexes of Cr(III), featuring 2,6-*bis*(imidazolyl)pyridine (ImPyIm) and 2-imidazolylpyridine (ImPy) ligands. The complex [Cr(ImPy)_3_]^3+^ displays luminescence at 803 nm on the microsecond time scale (13.7 μs) from a spin-flip doublet excited state, which transient absorption spectroscopy reveals to be populated within several picoseconds following photoexcitation. Conversely, [Cr(ImPyIm)_2_]^3+^ is nonemissive and has a ca. 500 ps excited-state lifetime.

## Abstract 2

The synthesis, characterization, and photophysical study of homoleptic N-heterocyclic carbene complexes of Cr(III) are provided, and near-infrared (NIR) spin-flip luminescence in fluid solution with microsecond lifetime is observed.

Photoactive complexes of Cr(III) have long been of interest due to long-lived luminescence in the deep-red and near-infrared (NIR) spectral regions, which originates from spin-flip metal-centered (^2^MC) excited states of doublet multiplicity (e.g., ^2^E and ^2^T_1_).^1−4^ While classical Cr(III)-centered luminophores have typically been plagued by low quantum yields (Φ_em_ < 0.1%),^5,6^ pioneering developments made in molecular design over the past decade, in particular the reporting of the efficient NIR emitter [Cr(ddpd)_2_]^3+^ (ddpd = *N*,*N*′-dimethyl-*N*,*N*′-dipyridine-2-ylpyridine-2,6-diamine),^7^ has allowed Cr(III)-based emitters to reach quantum yields of up to 30%^8^ with luminescence lifetimes on the millisecond time scale.^9,10^ Key to this success is the use of strong-field donors in conjunction with a close-to-ideal octahedral coordination geometry in achieving large ligand-field splitting. This enhanced splitting shifts strongly distorted ^4^T_2_ states to higher energy, preventing their population through back-intersystem crossing (bISC) and therefore minimizing nonradiative deactivation of the desirable and luminescent ^2^T_1_/^2^E states.

With the continued development of Cr(III)-based emitters comes the need to control the energy, and hence the color, of luminescence. To date, the vast majority of Cr(III) luminophores feature polypyridyl-based ligand architectures and typically emit within the narrow range of ca.720–780 nm.^4^ The energy of the phosphorescent ^2^MC excited states is strongly dependent upon the degree of interelectronic repulsion at the metal center and is hence governed by the nephelauxetic effect of the ligand set.^11^ Consequently, diversification of ligand design and the introduction of new, ideally strong-field, donor moieties to Cr(III) coordination chemistry is an essential tool in achieving control over the energy of luminescence. We have recently reported the luminescent chromium(III) triazolyl complex [Cr(btmp)_2_]^3+^ [btmp = 2,6-*bis*(4-phenyl-1,2,3-triazol-1-ylmethyl)pyridine], although phosphorescence falls within the more typical spectral region at λ_em_ = 760 nm.^12^ A series of complexes featuring 1,8-(*bis*-oxazolyl)carbazolide ligands were reported to emit over the range 813–845 nm in fluid solution,^13^ whereas the complex [Cr(bpi)_2_]^3+^ [bpi = 1,3-*bis*(2′-pyridylimino)isoindoline] displays weak room temperature (r.t.) luminescence centered at 950 nm.^14^ The homoleptic neutral cyclometalate [Cr(ppy)_3_] is also reported to be luminescent at 910 nm.^15^ Emission is successfully shifted into the NIR-II range through the use of a π-donating carbazolato fragment in [Cr(dpc)_2_]^+^ [dpc = 3,6-di-*tert*-butyl-1,8-di(pyridine-2-yl)carbazolato] (λ_em_ = 1067 nm), albeit only observable at cryogenic temperature.^16^

In an effort to not only optimize ligand-field strength but also continue to diversify the range of donors utilized within photoactive Cr(III) coordination complexes, *N*-heterocyclic carbenes (NHCs) are a new avenue to explore. As strong σ donors, NHCs are anticipated to cause particularly large ligand-field splitting and consequent destabilization of deleterious ^4^T_2_ excited states, thus promoting long-lived luminescence from the interconfigurational doublet states. Indeed, NHCs are now ubiquitous throughout transition-metal coordination chemistry and have been used to achieve favorable photophysical properties for complexes of Fe(II),^17^ Fe(III),^18^ Co(III),^19^ and Mn(IV).^20^ Surprisingly, NHCs have seldom been combined with Cr(III) centers,^21^ featuring only within heteroleptic complexes which catalyze the oligomerization of ethylene but for which no photophysical properties are reported.^22−25^

The imidazolium salts ImPyIm-H_2_ and PyIm-H (Scheme 1) were deprotonated with lithium *bis*(trimethylsilyl)amide (LiHMDS) at −40 °C in anhydrous tetrahydrofuran (THF) before the addition of a suspension of Cr^II^Cl_2_ in THF. Subsequent aerial oxidation in the presence of NH_4_PF_6_ afforded **1** and **2** as air- and moisture-stable yellow solids in yields of 23% and 56%, respectively. The magnetic susceptibilities of **1** and **2** were 3.82 and 3.90 μ_B_, respectively, consistent with that expected for d^3^ coordination complexes with a quartet ground-state electronic configuration (3.87 μ_B_).

**Scheme 1:**
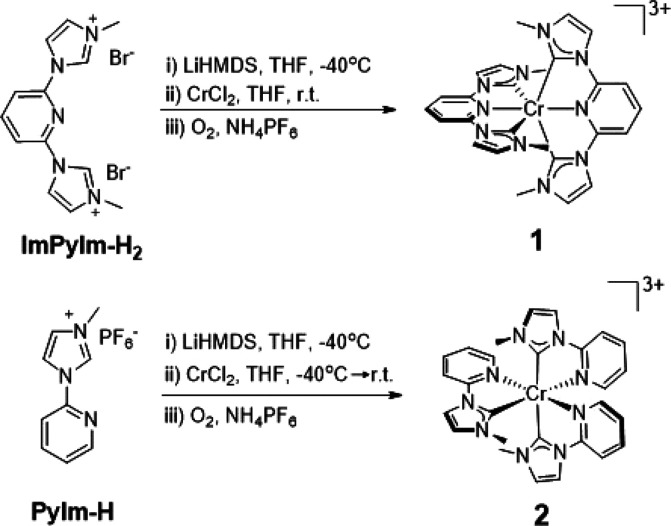
Synthetic Route to Cr(III) NHC Complexes **1** and **2**

X-ray diffraction studies reveal **1** to crystallize in space group *P*2_1_/*c* (Figure 1a), with C2–Cr1–C12 and C2–Cr1–N3 bond angles of 153.7(1)° and 76.7(1)°, respectively, revealing significant distortion of the coordination environment away from an ideal octahedral geometry due to the *tris*-chelating nature of the ligand. The torsion angle between the central pyridyl and flanking imidazolylidene moieties is 2.8°, revealing the essentially planar nature of the ligand, with the lack of conformational flexibility preventing helical wrapping around the Cr(III) center, as observed for [Cr(ddpd)_2_]^3+^ ^7^ and [Cr(btmp)_2_]^3+^ ^12^ for example. The Cr–N(pyridyl) bond length of 2.020(4) Å is typical for Cr(III) complexes,^7,10,12^ with the Cr–C(carbene) lengths being only slightly longer at ca. 2.1 Å. Complex **2** crystallizes in the *Pbcm* space group, with the molecular structure (Figure 1b) revealing that the asymmetric ligand adopts a meridional arrangement around the Cr(III) center. The use of *bis*-chelating ligands provides a pseudooctahedral coordination environment that is less distorted than that observed for **1**, with C9–Cr1–C9′ and C9–Cr1–N1 bond angles of 174.2(4)° and 77.9(2)°, respectively.

**Figure 1:**
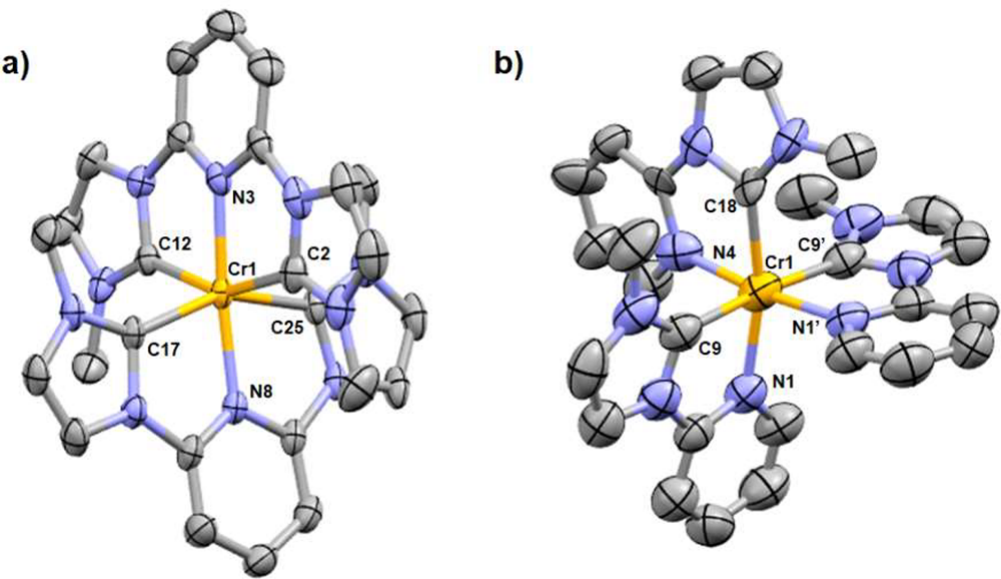
Molecular structures of **1** (a) and **2** (b). Thermal ellipsoids are shown at 50% probability, with H atoms, counterions, and cocrystallized solvent molecules removed for clarity.

**Figure 2:**
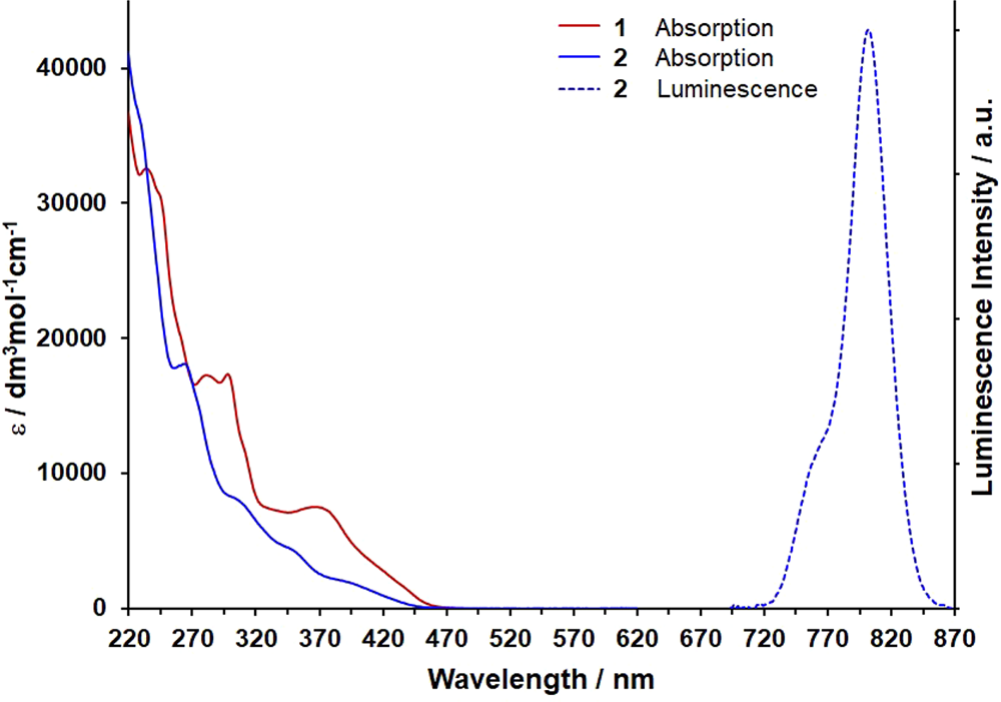
Electronic absorption and luminescence spectra (λ_ex_ = 350 nm) recorded for aerated r.t. MeCN solutions of **1** and **2**.

Electronic absorption spectra recorded for MeCN solutions of **1** and **2** are shown in Figure 2. Both complexes exhibit intense absorbance features in the region of 240 nm, attributed to ligand-localized π–π* excitations, with further transitions between 275 and 325 nm likely having largely ligand-based charge-transfer and π–π* character. With the aid of quantum-chemical calculations (Figures S11 and S12 and Tables S3 and S4), we assign the broad, moderately intense absorption envelope between 325 and 450 nm to transitions of predominantly ligand-to-metal charge-transfer (LMCT) character. These charge-transfer absorbances obscure very weak ligand-field absorptions, the lowest in energy of which are calculated to arise at approximately 25000 cm^–1^ for both **1** and **2**.

Cyclic voltammograms (Figure 3) show that both **1** and **2** display three fully electrochemically reversible reduction waves between −0.9 and −2.4 V versus ferrocenium/ferrocene (vs Fc^+^/Fc; Table 1). Because electrochemical reduction in complexes of Cr(III) may be metal-based^7^ or ligand-based,^26−28^ we carried out spectroelectrochemical monitoring to aid our assignment of each reduction process (Figures S2 and S3). Changes in the absorption spectra accompanying the first reduction process at ca. −0.9 V reveal the growth of new absorbances across the visible region, in particular the appearance of a broad, featureless band centered at 700 nm for **1** and two bands at 630 and 790 nm for **2**. The position and intensity of these bands (ε ≈ 8000 M^–1^ cm^–1^) are not compatible with those expected for a metal-localized reduction process^10,27^ but rather are more consistent with π–π* or charge-transfer transitions associated with coordinated ImPyIm^•–^ and ImPy^•–^ radical fragments. Further electrochemical reduction of **1** and **2** results in the appearance of additional significant features across the visible region (Figures S2 and S3), which are again consistent with ligand-localized transitions.

**Figure 3:**
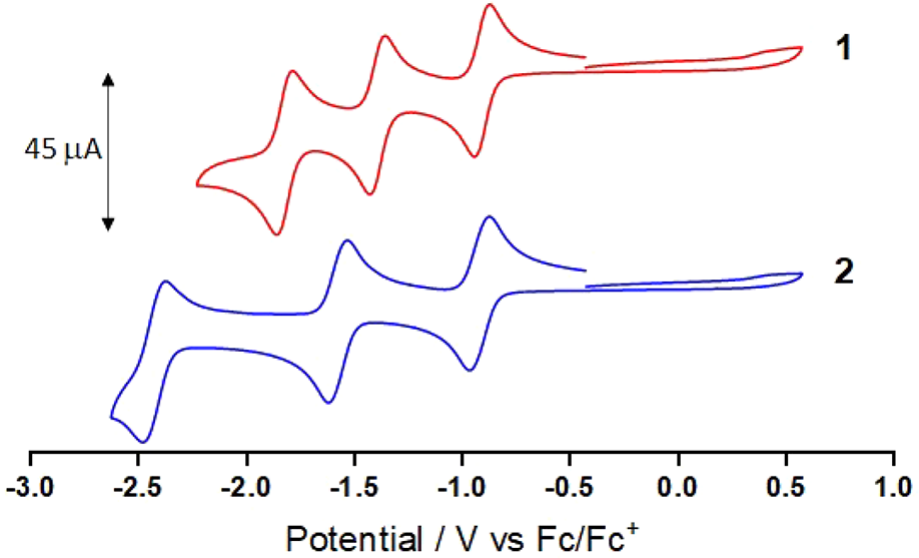
Cyclic voltammograms (vs Fc^+^/Fc) recorded at 100 mV s^–1^ for 1.5 × 10^–3^ mol dm^–3^ MeCN solutions of **1** and **2** containing ^*n*^Bu_4_NPF_6_.

**Table 1:** Summarized Photophysical and Electrochemical Data Recorded for MeCN Solutions of **1** and **2**

				*E*_1/2_/V vs Fc^+^/Fc (Δ*E*_a,c_/mV)^d^		
	λ_abs_/nm (ε/mol^–1^ dm^3^ cm^–1^)^a^	λ_em_/nm^a^^,^^c^	τ_em_/μs	*E*_red(1)_	*E*_red(2)_	*E*_red(3)_
****1****	233 (32740), 245 (30555), 284 (17220), 300 (17300), 370 (7475), 422 (2580)			–0.91 (72)	–1.39 (66)	–1.92 (70)
****2****	230 (35775), 237 (17720), 308 (7850), 351 (4300), 398 (1760)	803	13.7^a^ (18.8^b^)	–0.92 (81)	–1.58 (85)	–2.43 (94)

a Aerated MeCN.

b Deaerated MeCN.

c λ_ex_ = 350 nm.

d Recorded at 100 mV s^–1^. Δ*E*_a,c_ for Fc^+^/Fc = 70 mV.

Excitation of an aerated acetonitrile (MeCN) solution of **2** at 350 nm results in the observation of a sharp luminescence band centered at 803 nm accompanied by a less intense shoulder at 765 nm, which is absent from the spectrum recorded at 77 K (Figures 2 and S6). These spectral features are assigned to phosphorescence from the closely spaced and thermally equilibrated ^2^E and ^2^T_1_ excited states, respectively. Consistent with this assignment, a luminescence lifetime of 13.7 μs at r.t. was determined by time-correlated single photon counting (TCSPC), which increases to 18.8 μs upon exclusion of molecular oxygen. Likewise, the quantum yield of luminescence increases marginally for deoxygenated fluid solutions from 0.014 to 0.016%, which, although low, is respectable for complexes that emit beyond 800 nm under ambient conditions. This low luminescence efficiency may, in part, be due to a decrease in energy of the emissive states and consequent energy-gap law effects,^13,29,30^ while torsional degrees of freedom available within the bidentate ligands may result in a more flexible structure and consequent enhancement of nonradiative deactivation channels. The position of the observed luminescence maximum is noteworthy because it is shifted outside of the rather narrow range typically observed for the more extensively studied polypyridyl-based Cr(III)-centered spin-flip emitters, likely due to a subtly increased nephelauxetic effect imparted by the NHC donors. In stark contrast to **2**, complex **1** is nonemissive in both fluid solution and in a frozen solvent glass at 77 K. This is attributed to the significant deviation of the coordination sphere away from an ideal octahedral geometry upon coordination of the conformationally rigid ImPyIm ligand and the consequent reduction in the ligand-field strength despite the presence of four NHC donors.

Following excitation of an MeCN solution of **2** with a 400 nm, 40 fs laser pulse, a very broad transient signal appears across the spectral window (350–700 nm; Figure 4b), which undergoes complex evolution within the first 10 ps. Global analysis reveals several decay components (Figure S10), sub-100 fs, 0.38 ps, 2.3 ps, and a constant. The fastest, sub-100 fs process is too close to the instrument response to be resolved and likely corresponds to internal conversion within a hot manifold of quartet states, convolved with intersystem crossing (ISC) to the doublet manifold; surprisingly, ISC in **2** occurs much faster than the ∼800 fs that we recently determined for a luminescent Cr(III) complex.^12^ The spectrum of the next kinetic component, 0.38 ps, shows a bleaching of the ground-state absorbencies below 370 nm and, superimposed on a broad positive background, spectral features at 430, 550, and 630 nm, which somewhat resemble those of two-electron-reduced **2** (Figure S3b). With a lifetime of ca. 0.38 ps, this species evolves into the next excited state, lacking the 430 nm feature but retaining rather indistinct 550 and 665 nm bands, in addition to a band at ca. 368 nm. With ca. 2 ps lifetime, this state evolves into the final excited state, whose spectrum is dominated by a broad, somewhat structured absorption band centered at 430 nm (473 nm shoulder), with a weaker band at 615 nm and a small feature at 385 nm. This spectral shape persists beyond the time scale of the experiment (7 ns). The difference between the spectra associated with the 0.38 and 2.32 ps components (Figure S10d) implies population of states of different electronic origin within the doublet manifold. The final component represents population of the close-lying and thermally equilibrated phosphorescent ^2^T_1_ and ^2^E states. The lifetime of this final excited state determined by independent laser flash photolysis experiments (Figure S8) is 13.4 μs, being in good agreement with the emission lifetime determined by TCSPC measurements.

**Figure 4:**
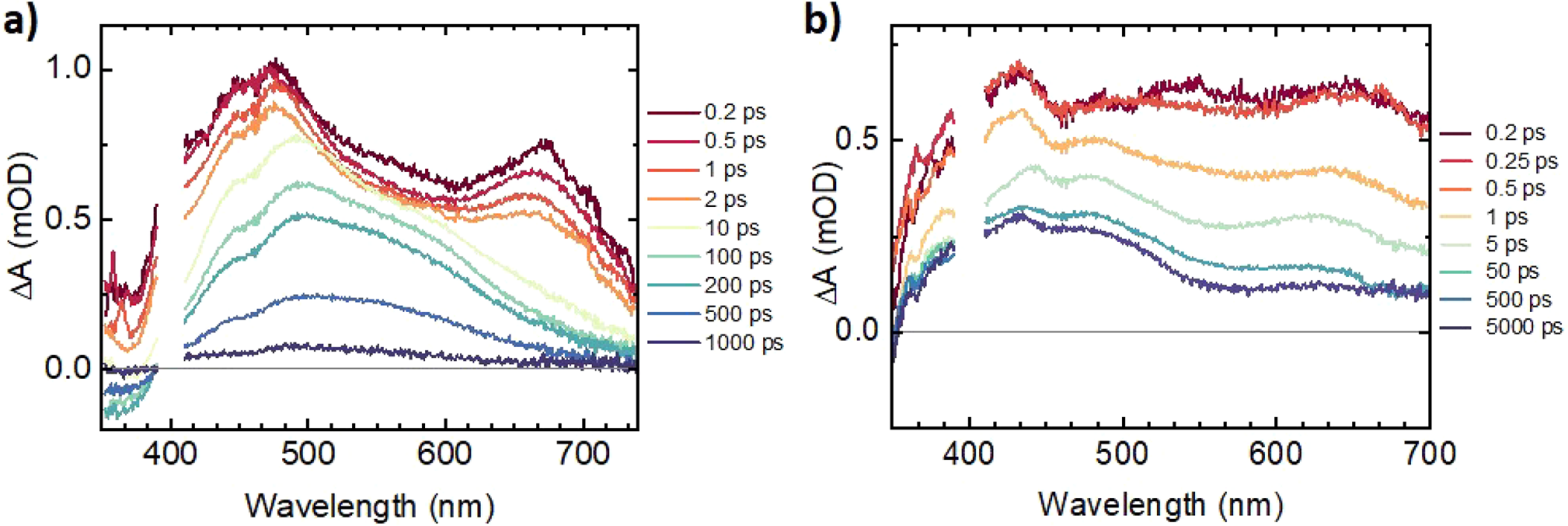
Transient absorption spectra recorded for aerated MeCN solutions of (a) **1** and (b) **2** (λ_ex_ = 400 nm).

Mirroring the stark differences in the photoluminescence characteristics of **1** and **2**, transient absorption spectra recorded for **1** (Figure 4a) reveal rapid excited-state decay, with all excited-state absorption features decaying within 1.5 ns. Interestingly, the best fit of the ultrafast data was achieved with a branched kinetic model, where the state initially populated upon photoexcitation rapidly (<100 fs) evolves to populate two independent excited states (Figure S9). A short-lived state (τ = 3.9 ps) is characterized by a slightly structured broad absorption band centered around 500 nm (similar to doubly reduced **1**; Figure S2b). The second excited state has absorbances at approximately 400, 470, and 670 nm (similar to the absorption spectrum of the anion of **1**; Figure S2a) and a lifetime of 476 ps. These features are possibly representative of ^4^LMCT and/or strongly Jahn–Teller distorted ^4^T_2_ levels, populated via ^2^MC-deactivating bISC (τ < 100 fs) as a result of the distorted geometry and weakened ligand field, the strength of which may also depend upon the relative arrangement of pyridyl and NHC fragments. Alongside the possible operation of additional nonradiative deactivation pathways, this rapid deactivation of the excited state accounts for the lack of spin-flip luminescence in **1**.

In summary, the first two examples of homoleptic Cr(III) complexes featuring NHC donors are presented. [Cr(ImPy)_3_]^3+^ displays spin-flip luminescence at 803 nm on the microsecond time scale, with the luminescent excited states populated within several picoseconds following photoexcitation. The employment of NHC donors provides sufficient ligand-field strength to promote population of the desirable doublet excited states, with minimal bISC to the deactivating quartet manifold. Continuing to widen the scope of donors employed within photoactive Cr(III) complexes will enable a significant expansion of chemical space for future exploration and the further development of new NIR-emissive materials.

## Supporting Information Available

Experimental methods and instrumentation, synthetic procedures, crystallographic data, steady-state and time-resolved photophysical data, and computational details and data (PDF)
