# Supplementary material for: A Near-Infrared Luminescent Cr(III) N-Heterocyclic Carbene Complex
Source: Inorg Chem. 2024 May 2;63(19):8526–30. doi: 10.1021/acs.inorgchem.4c01270 (PMC11094792; doi:10.1021/acs.inorgchem.4c01270)
Supplement: Supplementary file 1 — ic4c01270_si_001.pdf [file ic4c01270_si_001.pdf]

Supporting Information for

A Near Infra-Red Luminescent Cr(III) *N*-Heterocyclic Carbene Complex

Robert W. Jones,[a] Rory Cowin,[b] Iona I. Ivalo,[b] Dimitri Chekulaev,[b] Thomas M. Roseveare, [b] Craig. R. Rice,[a] Julia. A. Weinstein, [b] Paul. I. P. Elliott [a] and Paul A. Scattergood [a*]

[a] *Department of Chemistry, University of Huddersfield, Queensgate, Huddersfield, HD1 3DH, UK.*

[b] *Department of Chemistry, University of Sheffield, Brook Hill, Sheffield, S3 7HF, UK.*

Corresponding author: p.scattergood@hud.ac.uk

Contents

General methods and instrumentation		**S2**
Synthetic procedures and characterisation		**S4**
X-Ray crystallography instrumentation and methods		**S6**
Figure S1	Symmetry-related positional disorder in single crystals of complex **2**	**S7**
Table S1	Summary of X-Ray Crystallographic data for complexes **1** and **2**	**S7**
Table S2	Selected bond lengths and angles for crystal structures of **1** and **2**	**S8**
Figure S2	Spectroelectrochemical data for complex **1**	**S9**
Figure S3	Spectroelectrochemical data for complex **2**	**S10**
Figure S4	UV-Visible electronic absorption spectrum of pro-ligand **ImPyIm-H$_2$**	**S11**
Figure S5	UV-Visible electronic absorption spectrum of pro-ligand **ImPy-H**	**S11**
Figure S6	Photoluminescence spectrum for complex **2** recorded at 77K	**S12**
Figure S7	Excitation spectrum recorded for an aerated MeCN solution of **2**	**S12**
Figure S8	Flash photolysis data collected for an aerated MeCN solution of **2**	**S13**
Figure S9	Transient absorption spectra recorded for an aerated MeCN solution of **1**	**S14**
Figure S10	Transient absorption spectra recorded for an aerated MeCN solution of **2**	**S15**
Computational methods and details		**S16**
Figure S11	TDDFT-calculated electronic absorption spectrum for **1**	**S17**
Figure S12	TDDFT-calculated electronic absorption spectrum for **2**	**S17**
Table S3	Natural Transition Orbitals for selected electronic transitions within **1**	**S18**
Table S4	Natural Transition Orbitals for selected electronic transitions within **2**	**S22**
Coordinates for optimised ground state geometry of **1**		**S26**
Coordinates for optimised ground state geometry of **2**		**S27**
References		**S28**

General Methods and Instrumentation

Reagents and Synthesis: All reagents were purchased from Sigma-Aldrich, Acros Organics and Fluorochem and used as received. Anhydrous THF and MeCN were obtained by distillation from CaH_2, purged with dry N_2 for a period of at least 15 minutes and stored over 4Å molecular sieves under an atmosphere of dry N_2. All synthetic manipulations involving Cr(II) salts were carried out under an atmosphere of dry N_2 using standard Schlenk line techniques. The reagent $CrCl_2$ was handled and stored within an Argon-filled glovebox. Size-exclusion chromatography was performed under gravity using a fritted column of 35 mm diameter and 1000 mm length filled with Sephadex LH-20 resin which had previously been left to swell in 3:2 (*v/v*) MeOH/MeCN solution overnight before use.

Structural and Magnetic Characterisation: NMR spectra were acquired on a Bruker Ascend 400 MHz spectrometer, with chemical shifts being reported relative to the residual solvent signal (CD_3OD: ^{1}H δ 3.31, ^{13}C δ 49.00; CD_3CN: ^{1}H δ 1.94, ^{13}C δ 1.32, 118.26).[1] High-resolution mass spectrometry data were collected on an Agilent 6210 TOF instrument with a dual electrospray ionisation source. Infra-Red spectra were recorded on a Shimadzu IRSpirit FTIR spectrometer equipped with a QATR-S ATR accessory. Elemental microanalysis was performed at London Metropolitan University. Magnetic susceptibility measurements were performed by Evans' method,[2] using a co-axial NMR tube containing the paramagnetic analyte in a solution of d^3-MeCN (580 μL) and ^{t}BuOH (20 μL).

Photophysical and Electrochemical Analysis: UV-Visible electronic absorption spectra were recorded on an Agilent Cary-60 spectrometer with luminescence spectra recorded on Horiba Fluoromax-4 or Agilent Eclipse spectrometers. For data acquired on the Agilent Eclipse instrument, spectra were collected over 15 accumulations, applying 10-point adjacent averaging to reduce signal noise. Luminescence quantum yields are reported relative to $[Ru(bpy)_3]^{2+}$ in aerated MeCN solution (Φ = 1.8%), with all complexes being excited at a single wavelength of common optical density. Quantum yields are thus determined from the ratio of integrated peak areas, with an assumed experimental uncertainty of ±10 %. Luminescence lifetimes were determined by time-correlated single photon counting (TCSPC) on an Edinburgh Instruments mini-τ, equipped with a ps diode laser (404 nm, 56 ps). Cyclic voltammetry measurements were conducted for 1.5 $mol dm^{-3}$ solutions in dry MeCN under an atmosphere of N_2 using a glassy carbon working electrode, a Pt wire counter and Ag/AgCl reference. Solutions contained 0.2 $mol dm^{-3}$ $^{n}Bu_4NPF_6$ as a supporting electrolyte, with all potentials referenced against the Fc^+/Fc couple. Spectroelectrochemistry measurements were recorded on an Agilent Cary-60 spectrometer using a quartz cuvette with a path length of 0.5 mm (BASi). Inserted into the cuvette were a platinum gauze working electrode (0.5 mm thickness), a platinum counter and an Ag/AgCl reference electrode. Solutions, of typical concentration 60-70 μM, were prepared using dry MeCN and contained 0.2 $mol dm^{-3}$ $^{n}Bu_4NPF_6$. Solutions were sparged with dry N_2 *via* a plastic microcapillary and performed under an atmosphere of dry N_2. All potentials are referenced against the Fc^+/Fc couple. During measurements, the applied potential was incrementally increased only when no further spectral

changes were apparent. For reversible couples, the applied potential was incrementally reversed to ensure the complete recovery of spectra.

Transient Absorption Spectroscopy

Transient absorption experiments were performed at the Lord Porter Laser Laboratory at the University of Sheffield using a Helios system (HE-VIS-NIR-3200, Ultrafast Systems). A Ti:Sapphire regenerative amplifier (Spitfire ACE PA-40, Spectra-Physics) provides 800 nm pulses (40 fs FWHM, 10 kHz, 1.2 mJ). 400 nm pump pulses (2.5 kHz, 0.2μJ) were generated through frequency doubling of the amplifier fundamental. The pump was focused onto the sample to a beam diameter of approximately 190 μm. The white light probe continuum was generated using a sapphire crystal and a portion of the amplifier fundamental. The intensity of the probe light transmitted through the sample was measured using a CMOS camera, with a resolution of 1.5 nm. Prior to generation of the white light, the 800 nm pulses were passed through a computer controlled optical delay line (DDS300, Thorlabs), which provides up to 7 ns of pump-probe delay. The instrument response function was approximated to be 100 fs (FWHM), based on the temporal duration of the coherent artifact signal from neat acetonitrile.

Flash Photolysis

Samples in solution were excited at 355 nm using a nanosecond pulsed LOTIS TII laser. A Xe lamp was used to continuously probe the absorption of the sample before and after excitation. The light passing through the sample was focused through a monochromator, and then a photomultiplier and detector to compare the relative absorption before and after excitation at each wavelength. The initial voltage on the detector was normalised at each wavelength to account for the emission spectrum of the lamp and absorption spectra of the sample.

Synthetic Procedures and Characterisation

Synthesis of 2,6-*Bis*(1-methylimidazolium)pyridine dibromide (ImPyIm-H_2)

Following a procedure adapted from the literature[3]: A mixture of 2,6-dibromopyridine (2.00 g, 8.44 mmol) and 1-methylimidazole (2.08 g, 25.33 mmol) was heated to 165°C under an inert atmosphere for 16 h in a screw-capped thick-walled pressure tube. After cooling, the resulting black solid was triturated with dichloromethane (10 mL) until a fine suspension formed, then tetrahydrofuran (10 mL) was added. The suspension was filtered, and the solids washed twice with tetrahydrofuran (10 mL) before drying *in vacuo* to afford a brown solid. The crude solids were then suspended in warm ethanol (15 mL) and stirred vigorously for 5 minutes. The suspension was filtered and the collected solids washed twice with ethanol (5 mL) before being dried *in vacuo* to yield the title compound as a white powder (2.20 g, 65%). ^{1}H NMR (CD_3OD, 400 MHz): δ 4.14 (s, 6H), 7.89 (broad s, 2H), 8.16 (d, *J* = 8.0 Hz, 2H), 8.50 (t, *J* = 8.0 Hz, 1H), 8.61 (broad s, 2H), 10.30 (broad s, 2H). ^{13}C{^{1}H} NMR (CD_3OD, 101 MHz): δ 37.38, 115.61, 120.66, 126.28, 146.14, 147.18. HRMS (ESI) calc'd for $(C_{13}H_{15}N_5)^{2+}$: m/z = 120.5664. Found: m/z = 120.5662 $(M\text{-}2Br^-)^{2+}$.

Synthesis of 3-Methyl-1-(2-pyridyl)imidazolium hexafluorophosphate (PyIm-H)

Following a procedure adapted from the literature[4]: A mixture of 2-bromopyridine (4.00 g, 25.32 mmol) and 1-methylimidazole (2.29 g, 27.85 mmol) was heated to 160°C under an inert atmosphere for 40 h in a screw-capped thick-walled pressure tube. After cooling, dichloromethane (10 mL) was added to the residue. Addition of excess diethyl ether afforded a precipitate which was collected by filtration and washed twice with tetrahydrofuran (10 mL). The resulting brown solid was dissolved in water and precipitated as a hexafluorophosphate salt through addition of solid ammonium hexafluorophosphate (4.54 g, 27.85 mmol), being collected by filtration and washed twice with water (5 mL). The solids were then dissolved in 9:1 (*v/v*) dichloromethane:acetonitrile (5 mL) and re-precipitated through addition of diethyl ether to afford the title compound as a white solid (2.08 g, 27%). ^{1}H NMR (CD_3CN, 400 MHz): δ 3.96 (s, 3H), 7.54 (t, *J* = 1.7 Hz, 1H), 7.57 (dd, *J* = 4.9, 7.0 Hz, 1H), 7.72 (d, *J* = 8.1 Hz, 1H), 8.06 (t, *J* = 1.7 Hz, 1H), 8.10 (td, *J* = 1.7 Hz, 8.1 Hz, 1H), 8.59 (dd, *J* = 1.0, 4.8 Hz, 1H), 9.25 (s, 1H). ^{13}C{^{1}H} NMR (CD_3CN, 100 MHz): δ 37.45, 114.96, 120.20, 125.71, 126.36, 135.59, 141.47, 150.48. HRMS (ESI) calc'd for $(C_9H_{10}N_3)^+$: m/z = 160.0875. Found: m/z = 160.0879. $(M\text{-}PF_6)^+$.

Synthesis of Complex 1

Lithium *bis*(trimethylsilyl)amide (2.0 mL, 1 M sol. in THF, 2 mmol) was added, dropwise, to a suspension of **ImPyIm-H_2** (0.40 g, 1 mmol) in anhydrous tetrahydrofuran (30 mL) at -40°C. The mixture was then allowed to warm to r.t. with stirring for 3 h. A vigorously stirring suspension of $CrCl_2$ (59 mg, 0.48 mmol) in anhydrous tetrahydrofuran (10 mL) was added to the reaction mixture dropwise by cannula, resulting in a purple suspension that was stirred for 1 h. The reaction mixture was transferred

into an aerated acetonitrile solution of ammonium hexafluorophosphate (0.26 g, 1.57 mmol, 100 mL) and stirred for 30 minutes, resulting in an orange/brown suspension. The volume was reduced to approximately 25 mL, filtered through celite, then evaporated to dryness. The resulting brown residue was purified by size-exclusion column chromatography (Sephadex LH-20, 3:2 MeOH: MeCN) which after two passes afforded the title compound as a bright yellow solid (0.11 g, 23%). HRMS (ESI). Calc'd for $CrC_{26}H_{26}N_{10}P_2F_6$ (M-PF_6^-): m/z = 820.1031. Found: m/z = 820.1017. Anal. Calc'd for $CrC_{26}H_{26}N_{10}P_3F_{18}$ (%): C 32.34, H 2.71, N 14.51, found (%): C 32.49, H 2.84, N 13.60. IR (ATR): $\bar{\upsilon}$ / cm^{-1} = 3152 (w), 1640 (m), 1627 (m), 1589 (m), 1571 (w), 1493 (s), 1476 (m), 1411 (w), 1381 (vw), 1354 (vw), 1311 (m), 1301 (m), 1281 (m), 1251 (w), 1196 (vw), 1158 (w), 1126 (m), 1090 (w), 1058 (vw), 1034 (w), 1015 (vw), 1004 (m), 898 (m), 822 (vs), 800 (s), 779 (s), 741 (s), 731 (m), 713 (m), 685 (w), 665 (m), 612 (vw), 555 (vs).

Synthesis of Complex 2

Lithium *bis*(trimethylsilyl)amide (1.5 mL, 1 M sol. in THF, 1.5 mmol) was added, dropwise to a suspension of **PyIm-H** (0.46 g, 1.5 mmol) in anhydrous tetrahydrofuran (30 mL) at -40°C. The mixture was stirred for a further 1 h. at -40°C. A vigorously stirring suspension of $CrCl_2$ (58 mg, 0.47 mmol) in anhydrous tetrahydrofuran (10 mL) was then added to the reaction mixture dropwise, by cannula. The mixture was stirred for 2 h. while warming to r.t, resulting in a dark red suspension. The reaction mixture was transferred into an aerated acetonitrile solution of ammonium hexafluorophosphate (0.26 g, 1.57 mmol, 100 mL) and stirred for 30 minutes, resulting in a yellow suspension. The volume was reduced to approximately 25 mL, filtered through celite, then evaporated to dryness. The resulting orange/brown residue was purified by size-exclusion column chromatography (Sephadex LH-20 3:2 MeOH: MeCN) which after two passes afforded the title compound as a yellow solid (0.25 g, 56%). HRMS (ESI). Calc'd for $CrC_{27}H_{27}N_9$ (M-3PF_6^-): m/z = 176.3932. Found: m/z = 176.3935. Anal. Calc'd for $CrC_{27}H_{27}N_9P_3F_{18}$ (%): C 33.62, H 2.82, N 13.07, found (%): C 33.64, H 2.65, N 12.75. IR (ATR): $\bar{\upsilon}$ / cm^{-1} = 3157 (vw), 1622 (m), 1582 (w), 1489 (m), 1460 (m), 1414 (w), 1361 (vw), 1335 (m), 1254 (w), 1170 (w), 1133 (w), 1094 (w), 1061 (w), 1028 (w), 964 (w), 826 (vs), 771 (s), 741 (m), 731 (s), 674 (w), 651 (w), 614 (vw), 555 (s).

Single Crystal X-Ray Diffraction:

Single crystals of **1** were obtained from the slow vapour diffusion of diisopropylether into a concentrated MeCN solution containing a small quantity of NH_4BF_4. Diffraction data were collected under a stream of cold N_2 at 150 K on a Bruker D8 Venture diffractometer equipped with a graphite monochromated Mo(kα) radiation source. Solutions were generated using Patterson heavy atom or direct methods and fully refined by full-matrix least-squares on F^2 data using SHELXS-97 and SHELXL software respectively.[5] Absorption corrections were applied based upon multiple and symmetry-equivalent measurements using SADABS.[6] The structure contained a rotationally disordered tetrafluoroborate counter-anion and the fluorine atoms were modelled over two positions using the PART instruction during the least squares refinement. No other restraints were required. (CCDC2279805)

Single crystals of **2** were obtained from the slow vapour diffusion of diethyl ether into a concentrated acetone solution. Diffraction data were collected at 100 K (Oxford Cryosystems Cryostream) on a Bruker D8 Venture diffractometer equipped with a IµS Microfocus Cu-Kα sealed-tube source and a Photon 100 CMOS (Complementary Metal Oxide Sensor) detector with shutterless capability. Data were corrected for absorption using empirical methods (SADABS) based on symmetry-equivalent reflections and measurements at different azimuthal angles.[7-9] Structure solution was achieved by direct methods and the crystal structure was refined using full-matrix least-squares on F^2 data using SHELXL[10] within Olex2.[11] Non-hydrogen atoms were refined anisotropically. Hydrogen atoms were placed in calculated positions, refined to idealized geometries (riding model) and assigned a fixed isotropic displacement parameter. (CCDC2296861)

The asymmetric unit of the structure solution consists of half of complex **1**, resulting in a 2-fold rotation axis intersecting the central Cr atom and the centre of the chemical bond between pyridyl and NHC moieties within a ligand (the C14-N5 bond). This results in one of the three ligands being disordered due to the 2-fold rotation, with the other two ligands being symmetry related (Figure S1). The symmetry related positional disorder of one of the ligands was resolved by fixing the occupancy of the two positions to 50%, applying a part -1 function, constraining the rings to idealised geometries and applying restraints to normalise the thermal displacement of the atoms. Disorder of one of the PF_6 counter-ions was also observed. This was modelled with conventional two-part disorder with occupancies of 51.5(18) % and 48.5(18) % of part 1 and part 2, respectively. Summary details of the solution are outlined in Table S1.

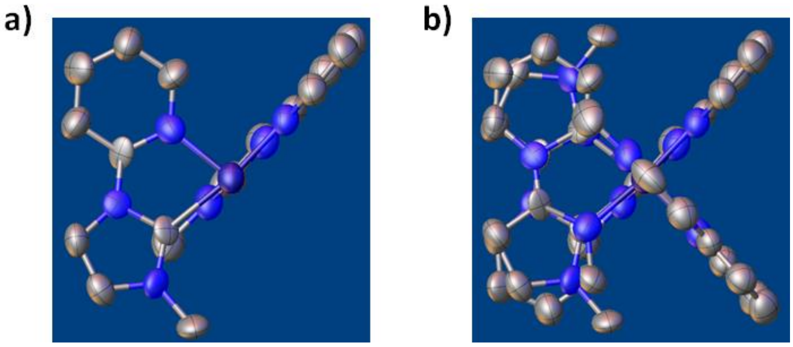

Figure S1 Image of the asymmetric unit (**a**) and the grown structure (**b**) demonstrating the symmetry related positional disorder of one of the three ligands. Images were created in Olex 2.

Table S1 Summary of crystallographic data for **1** and **2**.

**1**		**2**	
	$C_{30}H_{32}B_2CrF_{14}N_{12}P$		$C_{33}H_{39}CrF_{18}N_9O_2P_3$
Crystal colour	Yellow	Crystal colour	Clear Yellow
Crystal system	Monoclinic	Crystal size (mm)	0.14 × 0.06 × 0.04
Space group	$P2_1/c$	Crystal system	Orthorhombic
a (Å)	12.0676(6)	Space group	*P*bcm
b (Å)	13.0736(7)	*a* (Å)	10.6281(4)
c (Å)	24.5176(13)	*b* (Å)	17.5159(6)
α (°)	90	*c* (Å)	23.4340(9)
β (°)	91.312(2)	α (°)	90
γ (°)	90	β (°)	90
V (Å^3)	3867.1(3)	γ (°)	90
Density (g cm^{-3})	1.600	*V* (Å^3)	4362.5(3)
Temperature (K)	150	Density (g cm^{-3})	1.645
μ (mm^{-1})	0.447	Temperature (K)	100
Wavelength (Å)	0.71073	μ (mm^{-1})	4.315
Reflns. collected	35327	Wavelength (Å)	1.54178
Ind. reflns. [R_{int}]	9591 [0.0961]	2θ range (°)	8.32 to 130.782
Reflns. used in refinement, *n*	9591	Reflns. collected	139598
LS parameters, *p*	566	Ind. reflns. [R_{int}]	3833 [0.1791]
*R*1	0.0843	Reflns. used in refinement, *n*	3833
*wR*2	0.2297	LS parameters, *p*	404
S	1.032	Restraints, *r*	108
		$R1\ (F)^a\ I>2.0s(I)$	0.1077
		$wR2\ (F^2)^a$, all data	0.3481
		$S(F^2)^a$, all data	1.215

^{a} $=R1(F) = \Sigma(|F_o| - |F_c|)/\Sigma|F_o|$; $wR2(F^2) = [\Sigma w(F_o^2 - F_c^2)^2/\Sigma wF_o^4]^{1/2}$; $S(F^2) = [\Sigma w(F_o^2 - F_c^2)^2/(n + r - p)]^{1/2}$

Table S2 Selected bond lengths and angles for **1** and **2** as determined by X-ray crystallography.

<table>
<tr><th colspan="2">1</th><th colspan="2">2</th></tr>
<tr><td colspan="2">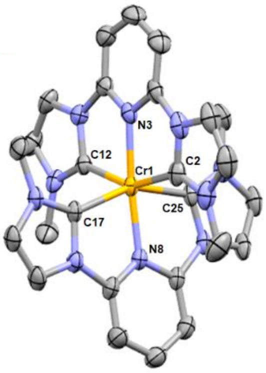
</td><td colspan="2">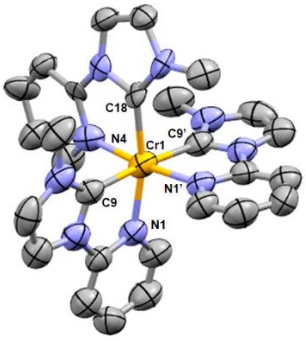
</td></tr>
<tr><td colspan="2">CCDC2279805</td><td colspan="2">CCDC2296861</td></tr>
<tr><td colspan="2">Lengths (Å)</td><td colspan="2">Lengths (Å)</td></tr>
<tr><td>Cr(1)-N(3)</td><td>2.020(4)</td><td>Cr(1)-N(1)</td><td>2.077(5)</td></tr>
<tr><td>Cr(1)-N(8)</td><td>2.022(4)</td><td>Cr(1)-N(4)</td><td>1.978(10)</td></tr>
<tr><td>Cr(1)-C(2)</td><td>2.095(4)</td><td>Cr(1)-C(9)</td><td>2.077(7)</td></tr>
<tr><td>Cr(1)-C(12)</td><td>2.106(4)</td><td>Cr(1)-C(18)</td><td>2.121(9)</td></tr>
<tr><td>Cr(1)-C(17)</td><td>2.093(4)</td><td></td><td></td></tr>
<tr><td>Cr(1)-C(25)</td><td>2.097(4)</td><td></td><td></td></tr>
<tr><td colspan="2">Angles (°)</td><td colspan="2">Angles (°)</td></tr>
<tr><td>N(3)-Cr(1)-N(8)</td><td>174.4(1)</td><td>N(1)-Cr(1)-C(18)</td><td>168.6(5)</td></tr>
<tr><td>C(2)-Cr(1)-C(12)</td><td>153.7(1)</td><td>C(9)-Cr(1)-C(9’)</td><td>174.2(4)</td></tr>
<tr><td>C(17)-Cr(1)-C(25)</td><td>153.7(1)</td><td>N(4)-Cr(1)-N(1’)</td><td>172.2(6)</td></tr>
<tr><td>N(3)-Cr(1)-C(12)</td><td>77.0(1)</td><td>N(1)-Cr(1)-N(4)</td><td>92.9(5)</td></tr>
<tr><td>C(2)-Cr(1)-C(25)</td><td>91.5(1)</td><td>N(4)-Cr(1)-C(9’)</td><td>96.9(7)</td></tr>
<tr><td>C(2)-Cr(1)-C(17)</td><td>93.8(1)</td><td>N(4)-Cr(1)-C(9)</td><td>87.6(6)</td></tr>
<tr><td></td><td></td><td>N(1)-Cr(1)-C(9)</td><td>77.9(2)</td></tr>
</table>

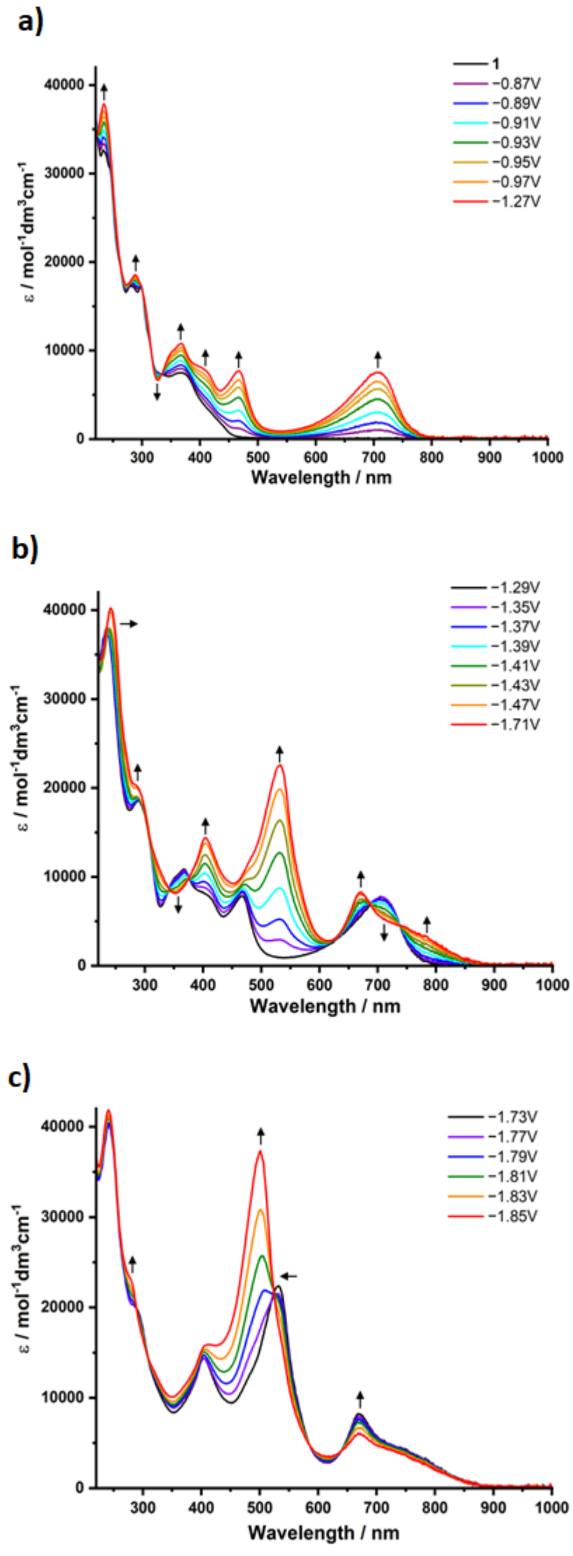

Figure S2 Changes in UV-Visible electronic absorption spectra accompanying the first (a), second (b) and third (c) electrochemical reduction processes of **1** in deaearted MeCN solution containing 0.2 moldm^{-3} $^{n}Bu_4NPF_6$ at r.t. All potentials are quoted relative to the Fc^+/Fc couple.

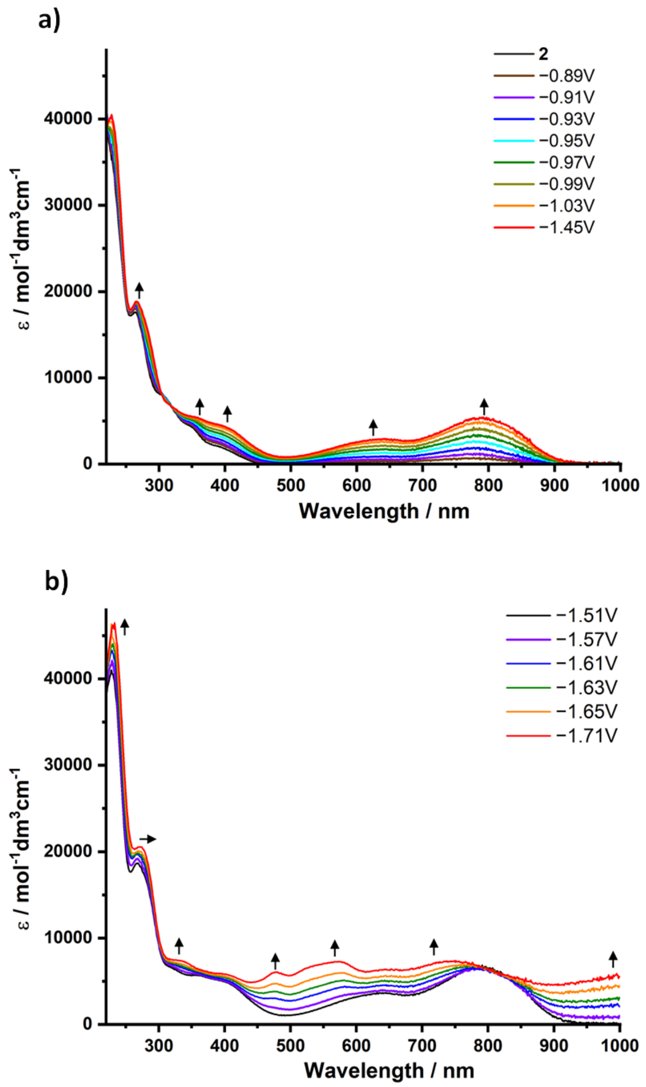

Figure S3 Changes in UV-Visible electronic absorption spectra accompanying the first (a) and second (b) electrochemical reduction processes of **2** in deaearted MeCN solution containing 0.2 mol dm^{-3} $^{n}Bu_4NPF_6$ at r.t. All potentials are quoted relative to the Fc^+/Fc couple. (Due to the cathodic nature of the third electrochemical couple we were unable to record satisfactory spectra associated with this process)

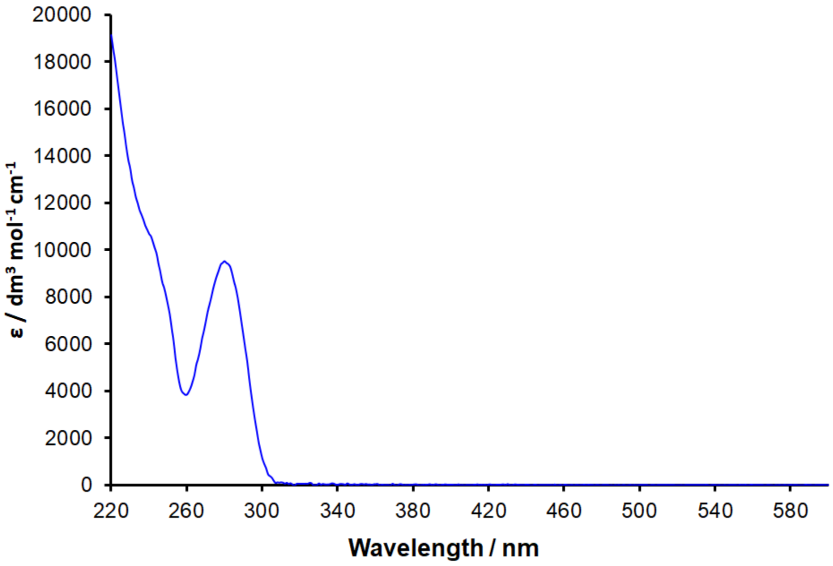

Figure S4 UV-Visible electronic absorption spectrum of the pro-ligand **ImPyIm-H_2** in MeCN

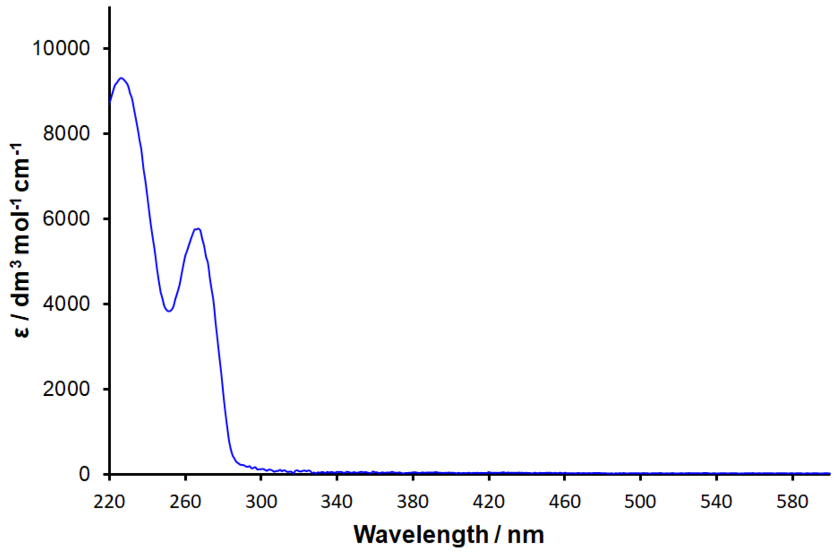

Figure S5 UV-Visible electronic absorption spectrum of the pro-ligand **ImPy-H** in MeCN

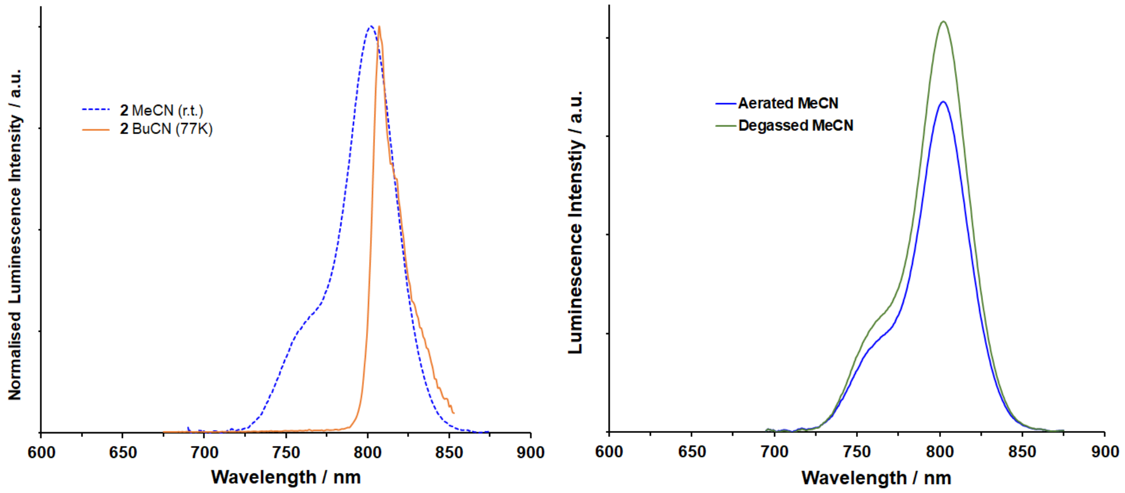

Figure S6 Left: Normalised luminescence spectra recorded for **2** in aerated MeCN solution at room temperature (dashed line) and at 77 K in frozen butyronitrile (solid line) (λ_{ex} = 350 nm). Right: Comparative luminescence spectra recorded for **2** in aerated (blue) and degassed (green) MeCN solutions at room temperature (λ_{ex} = 350 nm).

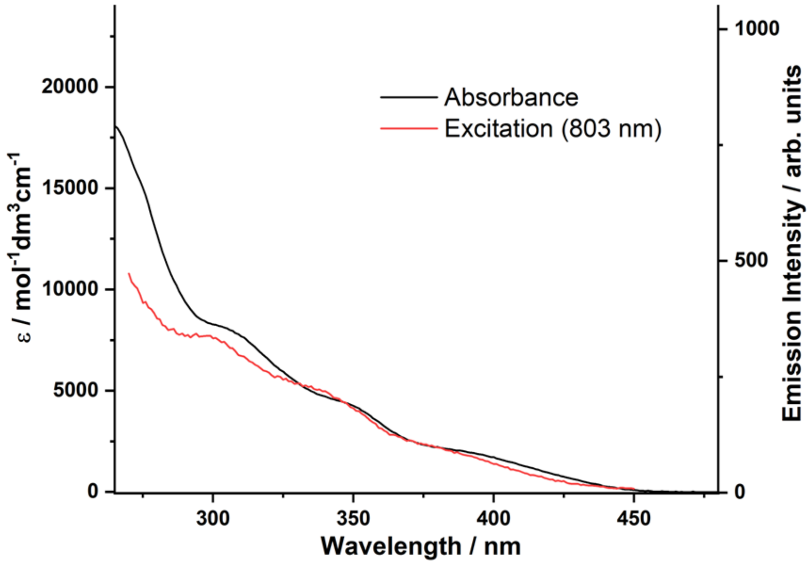

Figure S7 UV-Visible electronic absorption spectrum (black) and excitation spectrum (red) for luminescence at λ_{em} = 803 nm recorded for a solution of **2** in aerated MeCN at r.t.

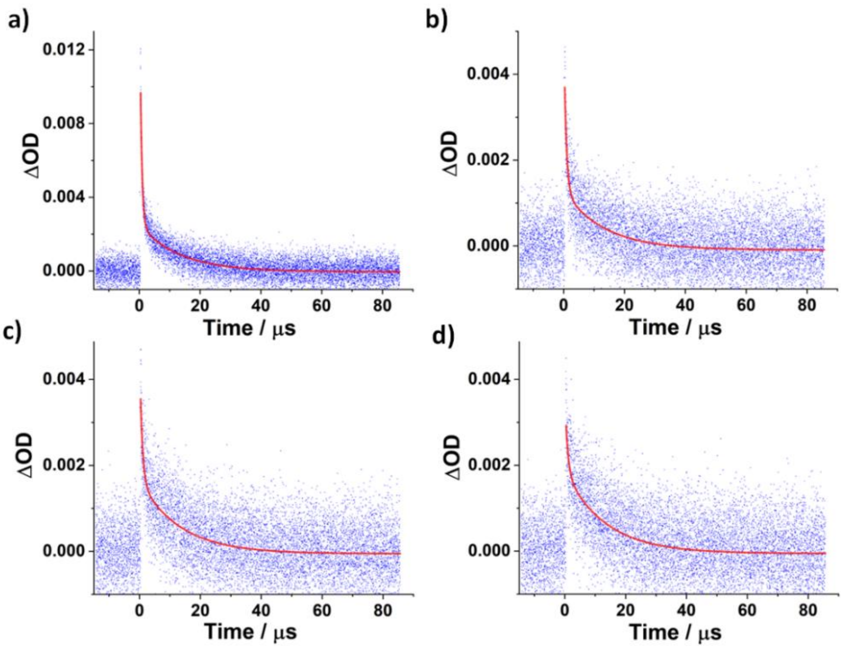

Figure S8 Representative flash photolysis data collected at 460 nm (**a**), 550 nm (**b**), 600 nm (**c**) and 615 nm (**d**) for an aerated MeCN solution of **2** following excitation at 355 nm. Decay traces are fitted with a geometric mean average lifetime which was found to be 13.40 ± 0.45 µs across the entire spectral range of 380-695 nm. This lifetime is in excellent agreement with the photoluminescence lifetime of 13.7 µs as determined by time correlated single photon counting, confirming that the long-lived species captured by both transient absorption spectroscopy and flash photolysis corresponds to the emissive $^{2}T_{1}/^{2}E$ metal-centred states. Fitting of flash photolysis decay traces required a second, very short component, with a mean average lifetime across the entire spectral range within the instrumental response function (20 ns) and so could not be satisfactorily resolved.

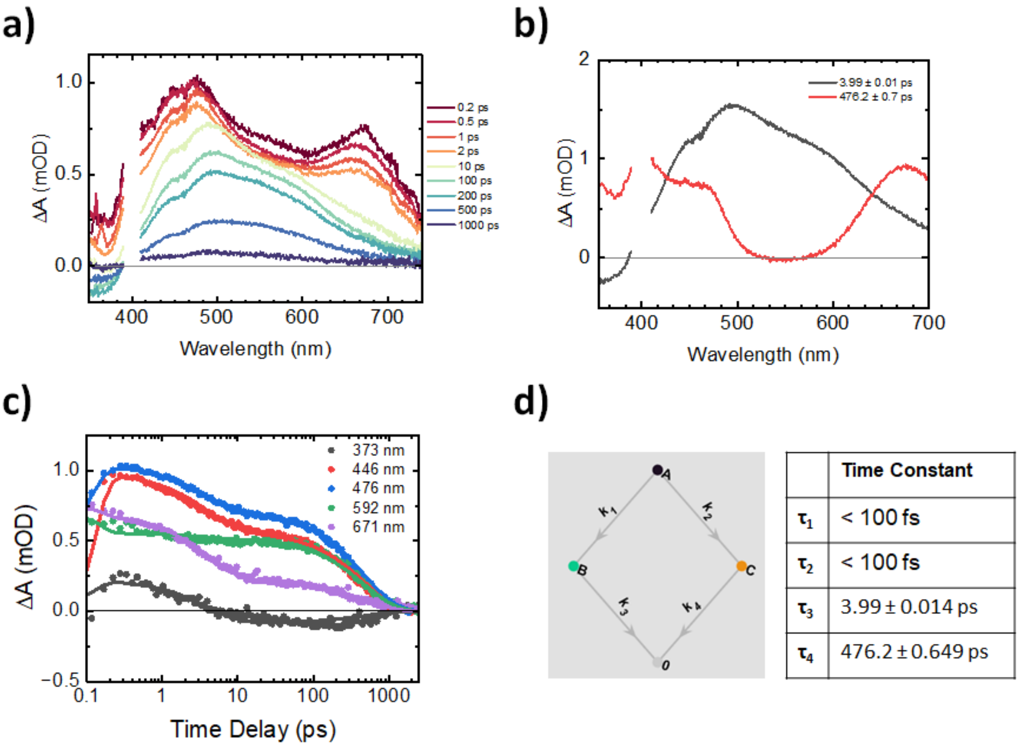

	Time Constant
τ_1	< 100 fs
τ_2	< 100 fs
τ_3	3.99 ± 0.014 ps
τ_4	476.2 ± 0.649 ps

Figure S9 **a)** Transient absorption spectra recorded for **1** in aerated acetonitrile solution (λ_{ex} = 400 nm), showing detail of transients recorded from 0.2 ps to 1 ns after excitation; **b)** decay-associated spectra (DAS) extracted from global analysis with time-constants of 3.99 and 476 ps; **c)** selected single-point kinetic traces obtained from global analysis; **d)** schematic of the branched kinetic model employed in the analysis of transient data and associated time-constants.

It is noted that excitation of **1** at either 400 nm or 375 nm produced the same results, with only those resulting from 400 nm excitation being shown here.

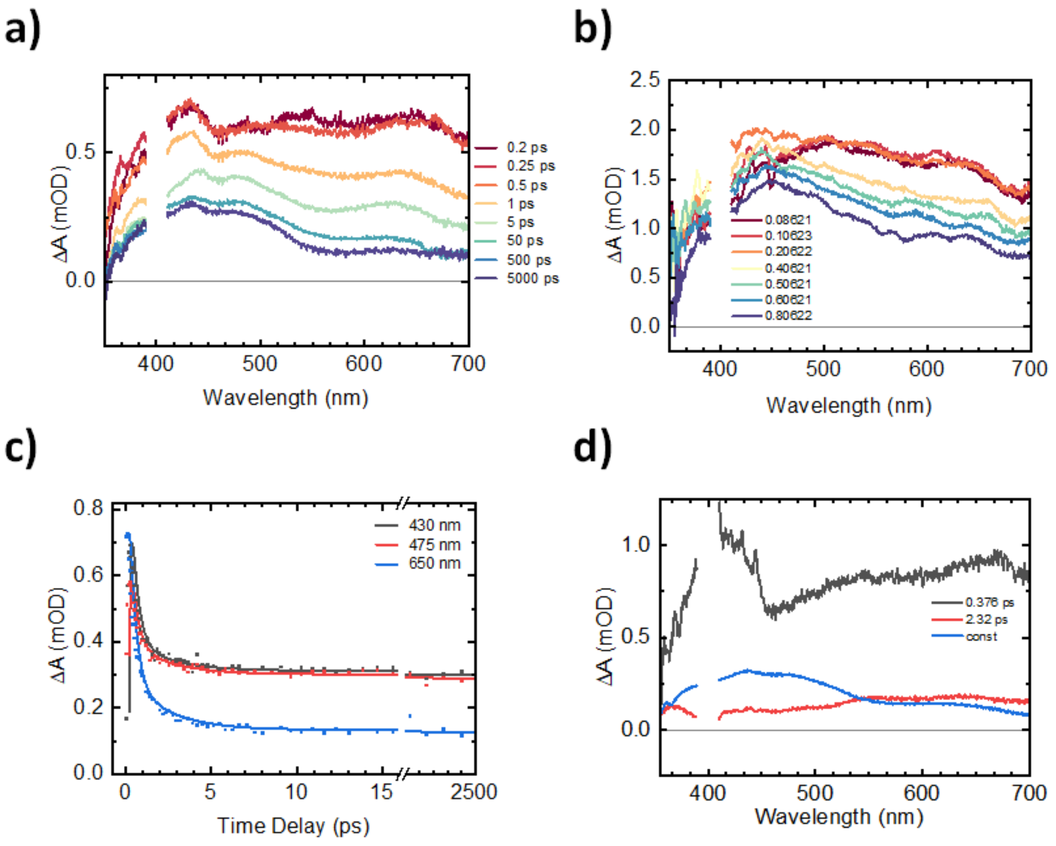

Figure S10 **a)** Transient absorption spectra recorded for **2** in aerated acetonitrile solution (λ_{ex} = 400 nm), showing detail of transients recorded from 0.2 ps to 5 ns after excitation; **b)** detail of transient absorption spectra recorded over early-times from 80 fs to 800 fs; **c)** selected single-point kinetic traces obtained from global analysis; **d)** decay associated spectra (DAS) extracted from global analysis. A sequential model of kinetic analysis yields four time constants: <100 fs (unresolved), 0.376±0.004 ps (τ_1), 2.32±0.03 ps (τ_2) and >7ns (τ_3, modelled as constant). The later component was independently determined to have a lifetime of 13.40 ± 0.45 μs by laser flash photolysis (see Figure S8).

It is noted that excitation of **2** at either 400 nm or 375 nm produced the same results, with only those resulting from 400 nm excitation being shown here.

Computational Methods and Details

The geometries of the ground states of complexes **1** and **2** were optimised using density functional theory (DFT) using the B3LYP hybrid functional[12, 13] (with 15 % Hartree-Fock, B3LYP*) with the def2-svp basis set[14] as implemented in the Orca 5.0 software package.[15, 16] All calculations were conducted using Grimme's D3-BJ dispersion correction[17, 18] along with the SMD implicit solvation model (acetonitrile).[19] In these DFT calculations the resolution-of-identity (RI) approximation for hybrid functionals (as implemented in ORCA) was employed to calculate the Coulomb energy term using the def2/j auxiliary basis set[20] and the exchange term by the so-called 'chain-of-spheres exchange' (COSX) algorithm. The geometries of the quartet ground states of the two complexes were optimised with location of minima confirmed through vibrational frequency analysis. Time-dependent DFT (TDDFT) was used to calculate the optical absorption spectrum using the lowest energy 100 excited states. Spin expectation values ($S^2 = S(S+1)$) for calculated excited states ranged from the ideal value for a quartet state of 3.75 to ~4.5 and hence a small degree of spin contamination is present for some states. Given that a quartet state can only be in equilibrium with a sextet state for which $S^2 = 8.75$, this small level of spin contamination was deemed acceptable for spin-allowed transitions. Natural transition orbitals (NTOs) were used to analyse the dominant character of the more intense transitions at $\lambda > 340$ nm. Molecular orbitals and natural transition orbitals were visualised using the Gabedit software package with isosurfaces set to 0.02.

Calculated spectra (Figures S11-S12) are broadly in agreement with experimental spectra. Analysis of natural transition orbitals (NTOs, Tables S3-S4) shows that for **2** the lowest energy transition and other lower energy transitions of greater oscillator strength are of ^{4}LMCT character which mask weaker Laporte forbidden ^{4}MC transitions. For **1** the two lowest energy transitions are of ^{4}MC state character and weak due to being Laporte forbidden. These again are masked by close-lying transitions of much greater intensity which are observed to have ^{4}LMCT state character.

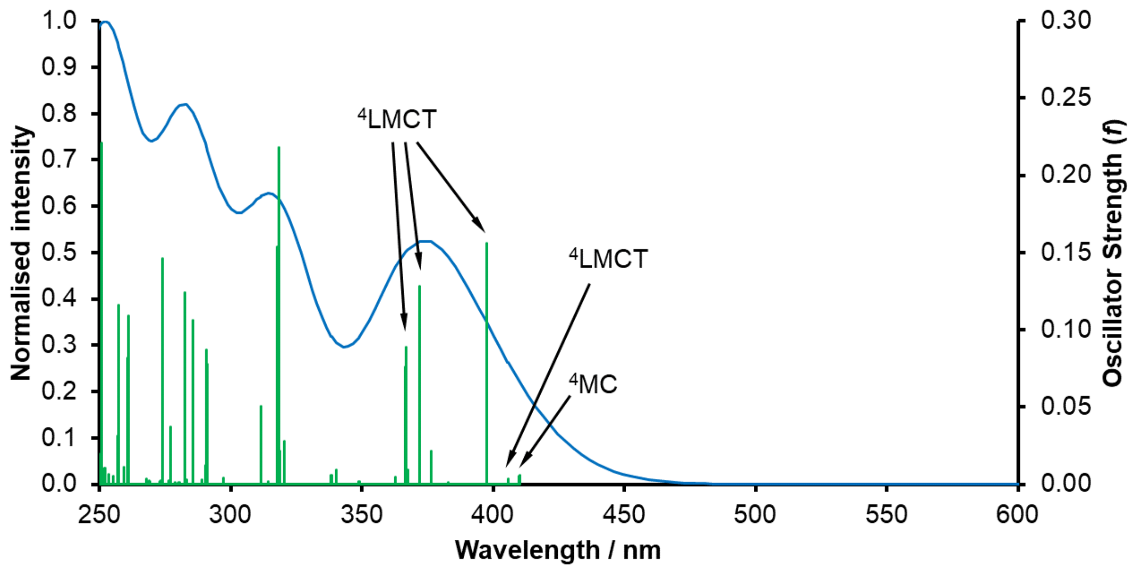

Figure S11 Calculated optical absorption spectrum for complex **1** showing positions of transitions and their oscillator strengths (green lines) and normalised convolution with 0.2 eV FWHM line broadening (blue trace).

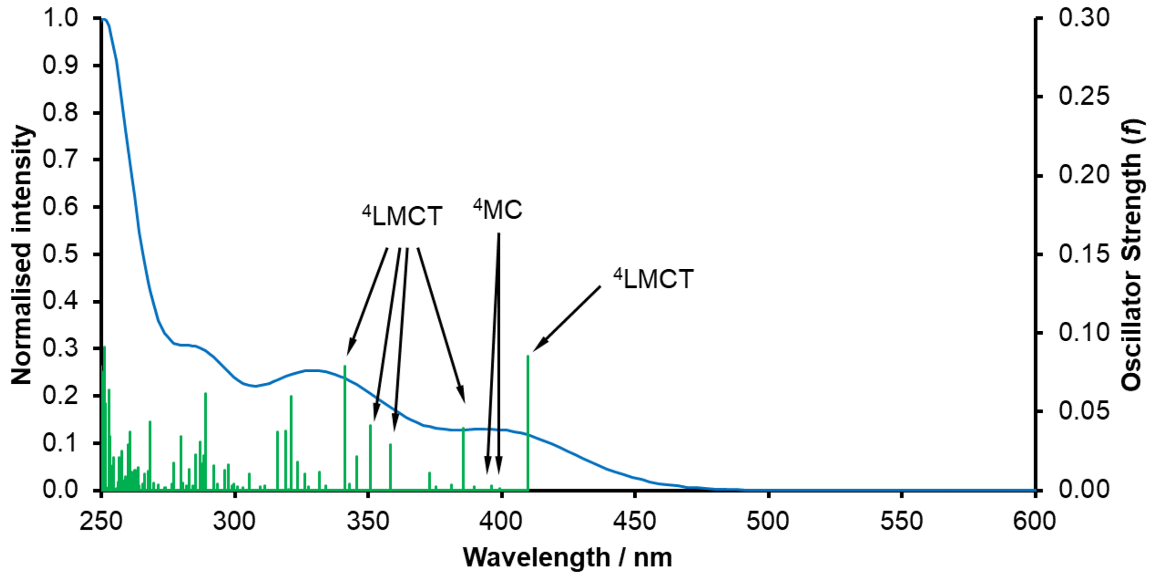

Figure S12 Calculated optical absorption spectrum for complex **2** showing positions of transitions and their oscillator strengths (green lines) and normalised convolution with 0.2 eV FWHM line broadening (blue trace).

Natural Transition Orbitals (NTOs)

Table S3 Natural transition donor (left) and acceptor (right) orbitals for selected optical transitions for complex **1**.

State 1: 24384.2 cm^{-1} 410.1 nm $S^2 = 3.91$

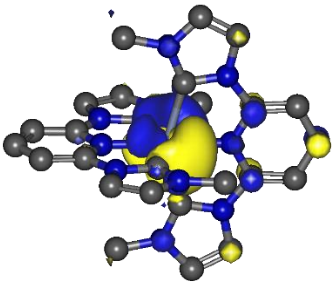
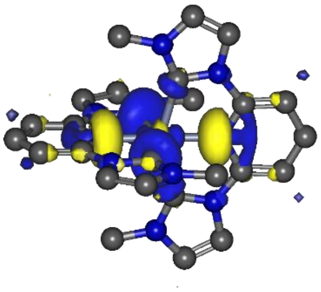

NTO 137α → NTO 138α 84.1 % ^{4}MC

State 2: 24405.9 cm^{-1} 409.7 nm $S^2 = 3.93$

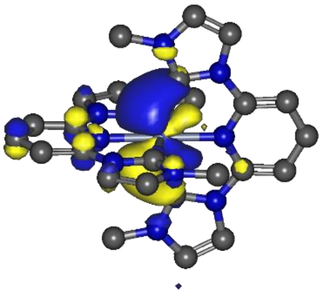
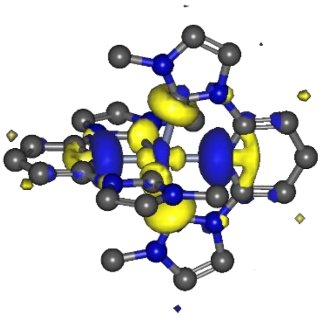

NTO 137α → NTO 138α 82.0 % ^{4}MC

State 3: 24651.3 cm^{-1} 405.7 nm $S^2 = 4.47$

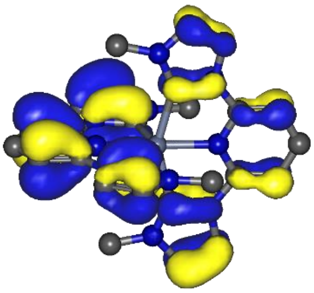
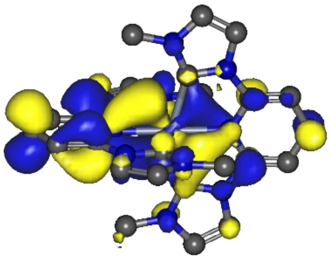

NTO 134β → NTO 135β 65.6 % ^{4}LMCT

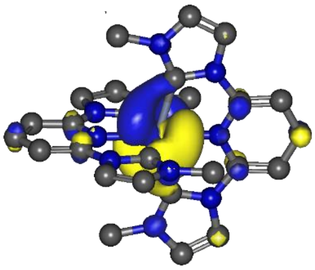
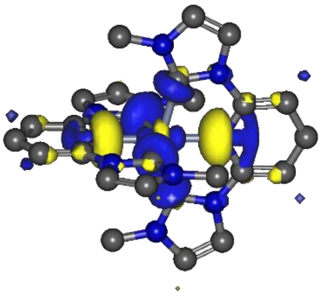

NTO 137α → NTO 138α 14.5 % ^{4}MC

State 4: 24660.1 cm^{-1} 405.5 nm S^2 = 4.46

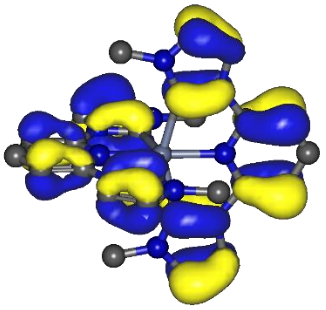 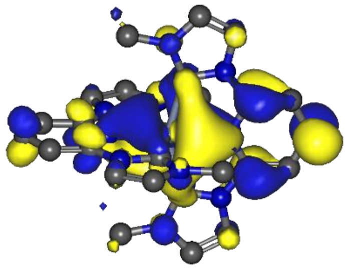

NTO 134β → NTO 135β 64.0 % ^{4}LMCT

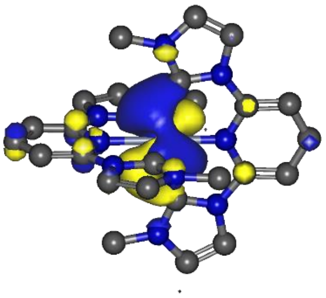 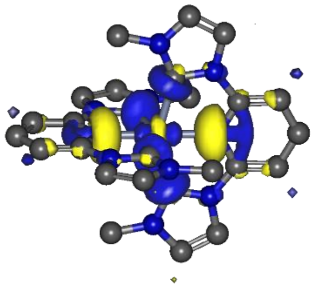

NTO 137α → NTO 138α 16.0 % ^{4}MC

State 5: 25165.2 cm^{-1} 397.4 nm S^2 = 4.49

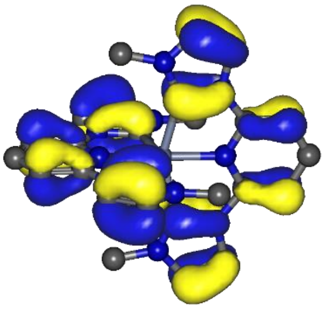 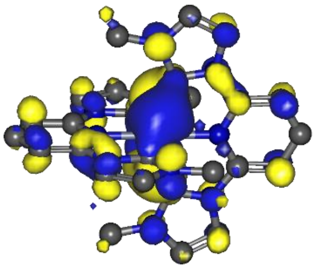

NTO 134β → NTO 135β 71.7 % ^{4}LMCT

State 9: 26881.0 cm^{-1} 372.0 nm $S^2 = 4.20$

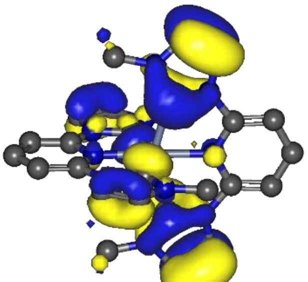
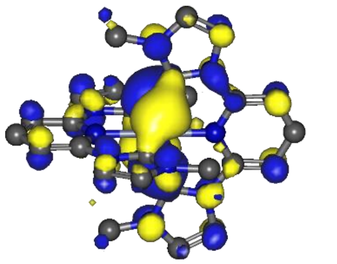

NTO 134β → NTO 135β 78.4 % ^{4}LMCT

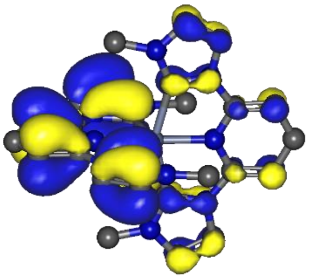
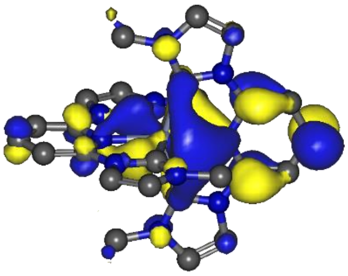

NTO 133β → NTO 136β 13.4 % ^{4}LMCT

State 10: 26899.4 cm^{-1} 371.8 nm $S^2 = 4.20$

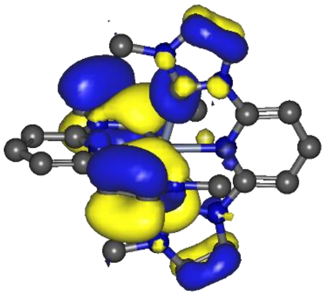
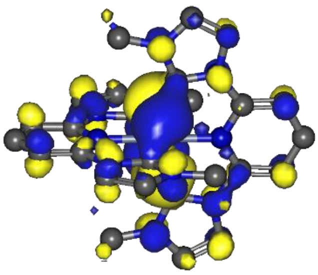

NTO 134β → NTO 135β 75.4 % ^{4}LMCT

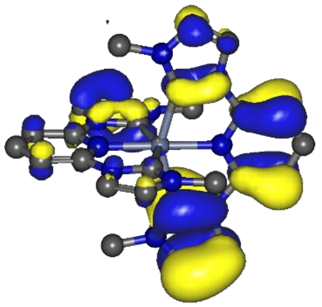
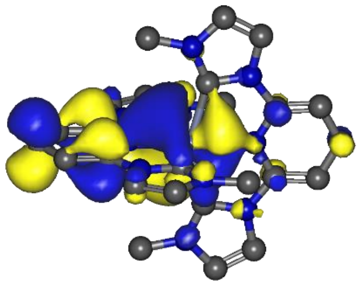

NTO 133β → NTO 136β 15.6 % ^{4}LMCT

State 13: 27267.3 cm^{-1} 366.7 nm $S^2 = 4.53$

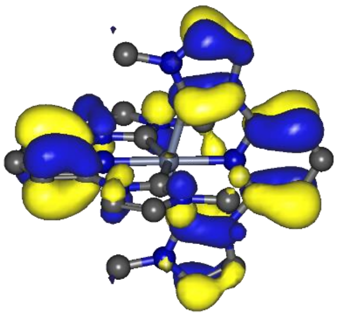

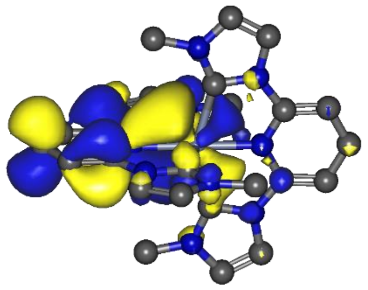

NTO 134β → NTO 135β 72.9 % ^{4}LMCT

State 14: 27285.0 cm^{-1} 366.5 cm $S^2 = 4.48$

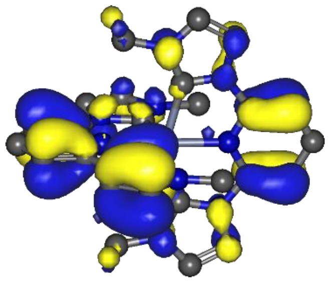

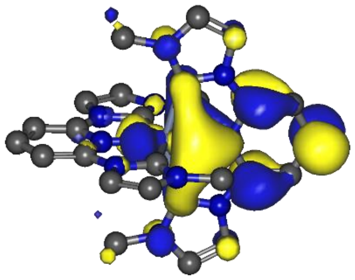

NTO 134β → NTO 135β 73.7 % ^{4}LMCT

Table S4 Natural transition donor (left) and acceptor (right) orbitals for selected optical transitions for complex **2**.

State 1: 24397.7 cm^{-1} 409.9 nm $S^2 = 4.38$

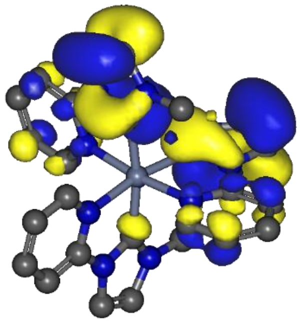

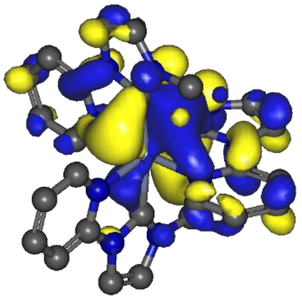

NTO 134β → NTO 135β 90.1 % ^{4}LMCT

State 2: 25053.1 cm^{-1} 399.2 nm $S^2 = 3.79$

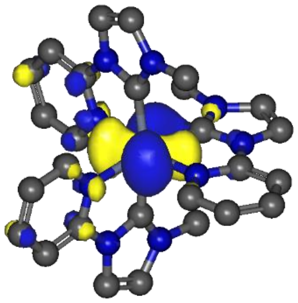

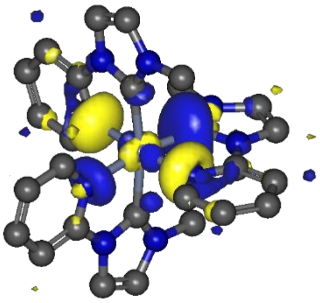

NTO 137α → NTO 138α 98.3 % ^{4}MC

State 3: 25247.8 cm^{-1} 396.1 nm $S^2 = 3.81$

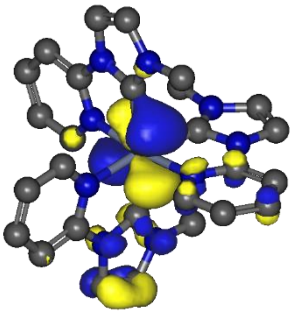

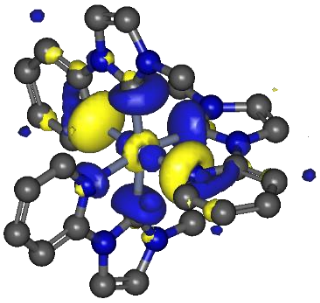

NTO 137α → NTO 138α 97.9 % ^{4}MC

State 4: 25661.5 cm^{-1} 389.7 nm $S^2 = 4.46$

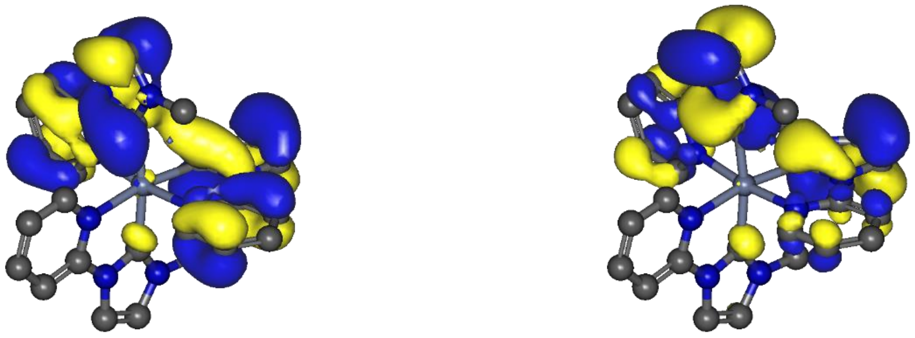

NTO 134β → NTO 135β 68.3 % ^{4}LMCT

State 5: 25925.1 cm^{-1} 385.7 nm $S^2 = 4.55$

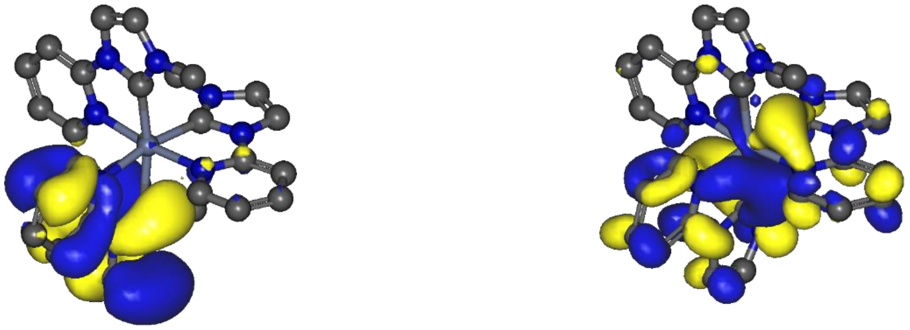

NTO 134β → NTO 135β 88.1 % ^{4}LMCT

State 9: 27924.1 cm^{-1} 358.1 nm $S^2 = 4.42$

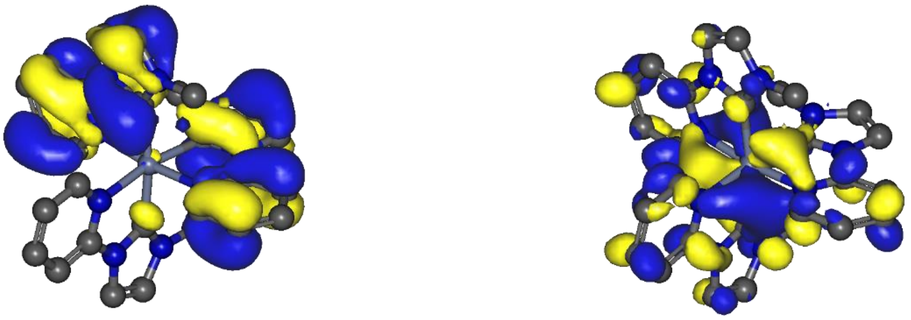

NTO 134β → NTO 135β 78.3 % ^{4}LMCT

State 10: 28507.8 cm^{-1} 350.8 nm S^2 = 4.45

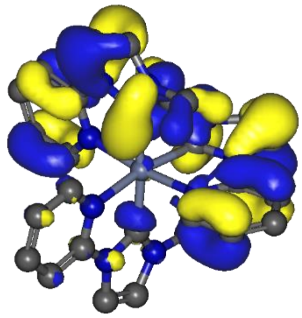
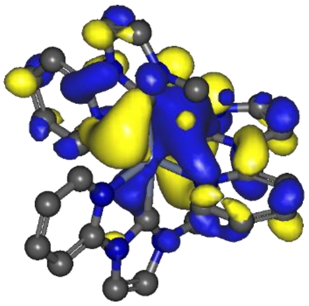

NTO 134β → NTO 135β 62.3 % ^{4}LMCT

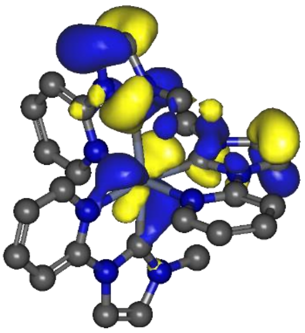
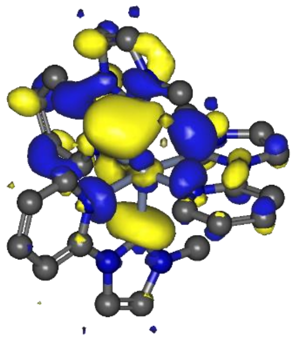

NTO 137α → NTO 138α 21.7 % ^{4}MC/^{4}LC

State 11: 28920.4 cm^{-1} 345.8 nm S^2 = 4.22

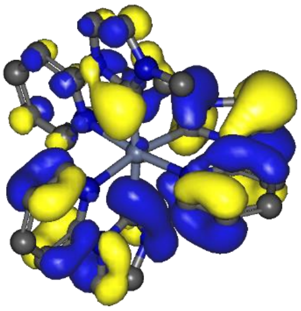
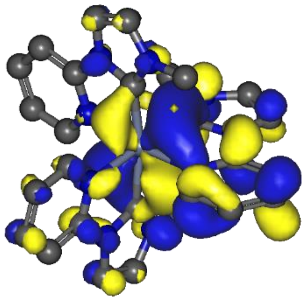

NTO 134β → NTO 135β 39.4 % ^{4}LMCT

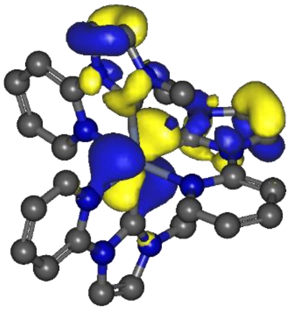
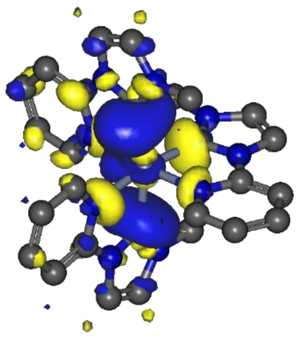

NTO 137α → NTO 138α 46.1 % ^{4}MC

State 13: 29316.0 cm^{-1} 341.1 nm $S^2 = 4.64$

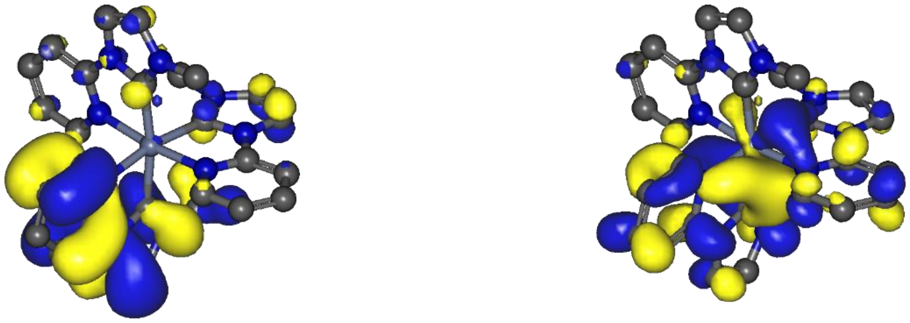

NTO 134β → NTO 135β 62.9 % ^{4}LMCT

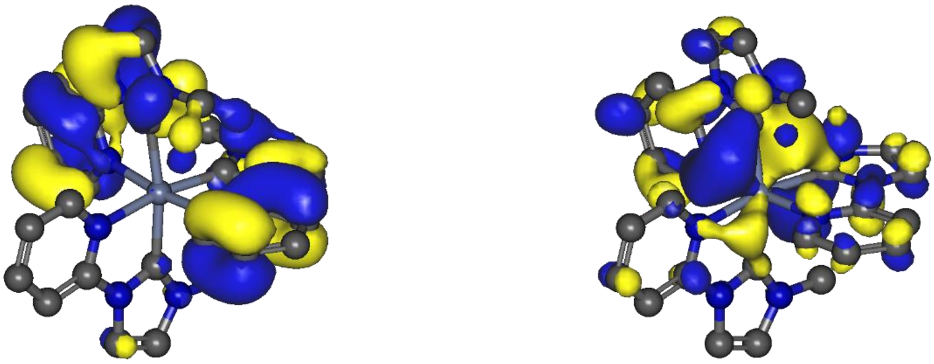

NTO 133β → NTO 136β 19.7 % ^{4}LMCT

Optimised Geometry (XYZ Coordinates) for Ground State of Complex **1**

63

Coordinates from ORCA-job gs

C	-0.94722566599858	0.16085414657908	-0.03505522311041
C	0.43367575497301	0.12441901908649	-0.20517887307495
C	1.15536891437884	1.26379274404820	0.16893684214740
C	0.51371280784898	2.39281240730038	0.69060734171923
C	-0.87144423222756	2.33529482869073	0.82187461143480
N	-1.57016948883551	1.24250302353087	0.46552442119317
N	-1.84402307232768	-0.87298267512861	-0.34199100411291
C	-3.18995816046854	-0.68168323125075	-0.11293098826623
N	-3.76741219036432	-1.82598876927525	-0.50685790635060
C	-2.81747649587724	-2.73324982445928	-0.97171512332076
C	-1.59778712873658	-2.13443363259140	-0.87176164814641
N	-1.69880489595064	3.35697633041603	1.31209128852145
C	-1.36617591645089	4.63179621115735	1.75712403526669
C	-2.54394966657486	5.21453067490995	2.11412002269425
N	-3.55632734057672	4.28526069391562	1.88052913198031
C	-3.05934955857827	3.14154510267809	1.38848809003487
C	-5.19601629456297	-2.10855027610625	-0.41634378324996
C	-4.96906733850287	4.55453258341358	2.11868234928089
C	-2.55289227109145	2.47606836451635	-2.76663827928046
C	-1.76776085627898	-0.02007155508391	3.87102731336274
C	-4.34718453329597	1.95350195809158	-1.13540875393764
N	-3.94472967582123	2.38788409162920	-2.33824241519986
C	-5.03399100132775	2.70066063205465	-3.14900114832553
C	-6.16325112275121	2.45969198712836	-2.42609076249991
N	-5.72392555278463	2.00080624562672	-1.18918982903163
C	-5.36089228216241	-0.11349778037005	4.31741980768044
C	-4.09411668936084	-0.31904812367092	4.77316930381809
N	-3.21923503899863	0.04004332521294	3.74920687856503
C	-3.88799004443167	0.46503444536302	2.66751334206650
N	-5.21800435328290	0.36888406423631	3.02140396601526
N	-5.67191517849616	1.18452083505668	0.93974015091164
C	-6.46306321899993	1.60381119226135	-0.06425802709205
C	-7.85016686801241	1.61579422691039	0.05311815247933
C	-8.39410609629664	1.16873478396354	1.26283927885709
C	-7.57682252628131	0.72894255701831	2.31035769851244
C	-6.20091180076471	0.75727019592805	2.09980820545203
Cr	-3.62061980137223	1.21833452744497	0.70417547315304
H	0.92923942456154	-0.75798688970673	-0.61256369765530
H	2.24246998104622	1.27214472784804	0.05093739392145
H	1.07305167479151	3.28286713138526	0.98346961497044
H	-3.09695961850057	-3.72054161045238	-1.33600523682859
H	-0.60159129939051	-2.48730682277609	-1.13078528040527
H	-0.34416812936210	5.00413940067464	1.78308520848562
H	-2.75555633443815	6.20495188363568	2.51422308914206
H	-5.46437490716847	-2.84780277770633	-1.18335353092518
H	-5.75904051638230	-1.18274764753694	-0.58858587593047
H	-5.43890062094328	-2.50863332345002	0.58091721832792
H	-5.53323248007338	3.62201431097978	2.00498390692926
H	-5.33464657658765	5.29786916630077	1.39294744251609
H	-5.10153547522046	4.94402240563908	3.13898612037519
H	-2.46016419342348	3.26829107975448	-3.52245404360468
H	-2.23052770650246	1.51566172740358	-3.19953881188972
H	-1.92417712041181	2.71954096759513	-1.90130542140975
H	-1.32072925176432	0.16706066776530	2.88829407100655
H	-1.46989112866862	-1.01684093270995	4.22896326826237
H	-1.42169706078984	0.74337313226434	4.58532798377424
H	-4.90603521677122	3.07090328997707	-4.16497557234633
H	-7.21566486841854	2.57521387750956	-2.67735211474028
H	-6.32847899909827	-0.26798323084297	4.79063328862080
H	-3.73445781089370	-0.68990318576553	5.73168081884332
H	-8.48346001420904	1.96069969933202	-0.76584271456861
H	-9.47996918919115	1.16306184803595	1.39212344397992
H	-7.99729368154557	0.38083577461363	3.25499549100148

Optimised Geometry (XYZ Coordinates) for Ground State of Complex **2**

64
Coordinates from ORCA-job gs

```
Cr -0.03476920860102 0.01075290295794 0.00546963418960
N 1.46808625697602 0.66247059673575 -1.37167583049029
N 1.06287001206268 -1.41456361027240 -2.30445534131319
N -0.35465019315362 -2.84271739002352 -1.53756331232500
N -1.63498761581908 0.76677539783348 -1.11042219755324
C -1.52043772261162 1.57337520018738 -2.18238917693380
H -0.50882477121960 1.86163640516276 -2.47437627349584
C -2.63407150463761 2.02797697480355 -2.88048430863416
H -2.50051701284995 2.68235896846643 -3.74486263449583
C -3.90514192282033 1.63269374631384 -2.45137407373801
H -4.80116217289419 1.97228309910733 -2.97867775452949
C -4.03123859155065 0.80071623092701 -1.33810950639456
H -5.00898081340898 0.47935105167286 -0.97566100282917
C -2.86431834590879 0.39212636468865 -0.69689167841678
C 2.11134555329507 1.84377183612001 -1.30760305491878
H 1.81303070057206 2.51804525429386 -0.50098385996397
C 3.10142737350050 2.19442336134943 -2.22012668248722
H 3.59972868408858 3.16290221679120 -2.13560829886143
C 3.43377954015609 1.28594874304666 -3.22980913112343
H 4.20704685819982 1.52869532690875 -3.96427273206054
C 2.77407853689537 0.05780252496053 -3.30107801638469
H 3.01563275622405 -0.67036088507693 -4.07678017194572
C 1.79597950007191 -0.21162446134448 -2.34534877254613
C 1.13383772391177 -2.51439176706594 -3.15092988549738
H 1.79887840110740 -2.56968886274342 -4.00987458624446
C 0.23435524787782 -3.41391585385240 -2.66035903757785
H -0.03731875660957 -4.40855188526956 -3.01005134528999
C -1.36839189688041 -3.50210251633022 -0.71767411226150
H -2.32936140751477 -2.97422548029046 -0.80279825972016
H -1.04430632975797 -3.51706756322595 0.33175609903277
H -1.48980400904888 -4.53358712680095 -1.07361597904030
C 0.14099644662550 -1.61499674785364 -1.30901767893633
C -0.99503021880858 -2.07928107737332 3.02154364558108
H -0.29130335618254 -2.72831919794808 2.48084782196544
H -1.54129371974595 -2.67593432720826 3.76346264182190
H -0.44224264393732 -1.27782897591312 3.53271170649840
C -1.64600468830323 -0.74572599429471 1.02496971545317
N -1.95962831026139 -1.50382329743133 2.08818175962935
C -3.33688346972769 -1.67460250063215 2.18143510632675
H -3.78859481817263 -2.26270021127587 2.97862238786563
C -3.90581464434531 -0.99449904629192 1.14526226300771
H -4.94756696908649 -0.86855848490543 0.85874695980432
N -2.84740373826167 -0.42730829797028 0.44567607662168
N 1.45973232394200 -0.63889478738687 1.32604456637483
N 1.07832684453850 1.42904835976042 2.28739212447876
N -0.37304130176015 2.84952632664552 1.57102731222927
C 2.09303379737137 -1.82554934248480 1.24649078925666
H 1.77846824658932 -2.49577059301522 0.44391901756375
C 3.09241284920134 -2.18907722707521 2.14240418245334
H 3.57984493937207 -3.16172659306180 2.04405656197530
C 3.44719949470620 -1.29084375540712 3.15356755881557
H 4.22827979476546 -1.54520045021304 3.87566172627176
C 2.79802889426625 -0.05880925407636 3.24198407178453
H 3.05421440531501 0.66232506641139 4.01960348956320
C 1.80932824558432 0.22638874489258 2.30219922837936
C 1.16535374617354 2.52393788244625 3.13873982902021
H 1.85352433428193 2.57833575843993 3.97928704441446
C 0.24501384495757 3.41885757935639 2.67932623621054
H -0.02546328754862 4.40862129503718 3.04352566696215
C -1.41413680638699 3.50608585643407 0.78396335333216
H -2.31454936060820 2.87686307904430 0.75214208723266
H -1.05364297506351 3.68887849407822 -0.23878623688572
H -1.65907015032281 4.46517531784585 1.25886633645598
C 0.12776037418059 1.62841998339527 1.31909180732268
```

References

1. G. R. Fulmer, A. J. M. Miller, N. H. Sherden, H. E. Gottlieb, A. Nudelman, B. M. Stoltz, J. E. Bercaw and K. I. Goldberg, NMR Chemical Shifts of Trace Impurities: Common Laboratory Solvents, Organics, and Gases in Deuterated Solvents Relevant to the Organometallic Chemist, *Organometallics*, 2010, **29**, 2176-2179.
2. E. M. Schubert, Utilizing the Evans method with a superconducting NMR spectrometer in the undergraduate laboratory, *Journal of Chemical Education*, 1992, **69**, 62.
3. D. S. McGuinness, J. A. Suttil, M. G. Gardiner and N. W. Davies, Ethylene Oligomerization with Cr−NHC Catalysts: Further Insights into the Extended Metallacycle Mechanism of Chain Growth, *Organometallics*, 2008, **27**, 4238-4247.
4. X.-B. Lan, Z. Ye, M. Huang, J. Liu, Y. Liu and Z. Ke, Nonbifunctional Outer-Sphere Strategy Achieved Highly Active α-Alkylation of Ketones with Alcohols by N-Heterocyclic Carbene Manganese (NHC-Mn), *Organic Letters*, 2019, **21**, 8065-8070.
5. , SHELXTL Program System.*Journal*, 1998.
6. G. M. Sheldrick, SADABS: A Program for Absorption Correction with the Siemens SMART System.*Journal*, 1996.
7. G. M. Sheldrick, *SADABS, Empirical Absorption Correction Program, University of Göttingen*, 1995, based on the methods of Blessing.
8. L. Krause, R. Herbst-Irmer, G. M. Sheldrick and D. Stalke, *J. Appl. Crystallogr.*, 2015, **48**, 3-10.
9. R. H. Blessing, *Acta Crystallogr. Sect. A Found. Adv.*, 1995, **A51**, 33-38.
10. G. M. Sheldrick, *Acta Crystallogr. Sect. C Struct. Chem.*, 2015, **71**, 3-8.
11. O. V. Dolomanov, L. J. Bourhis, R. J. Gildea, J. A. K. Howard and H. Puschmann, *J. Appl. Crystallogr.*, 2009, **42**, 339-341.
12. A. D. Becke, A new mixing of Hartree–Fock and local density-functional theories, *The Journal of Chemical Physics*, 1993, **98**, 1372-1377.
13. C. Lee, W. Yang and R. G. Parr, Development of the Colle-Salvetti correlation-energy formula into a functional of the electron density, *Physical Review B*, 1988, **37**, 785-789.
14. F. Weigend and R. Ahlrichs, Balanced basis sets of split valence, triple zeta valence and quadruple zeta valence quality for H to Rn: Design and assessment of accuracy, *Physical Chemistry Chemical Physics*, 2005, **7**, 3297-3305.
15. F. Neese, The ORCA program system, *WIREs Computational Molecular Science*, 2012, **2**, 73-78.
16. F. Neese, Software update: the ORCA program system, version 4.0, *WIREs Computational Molecular Science*, 2018, **8**, e1327.
17. S. Grimme, J. Antony, S. Ehrlich and H. Krieg, A consistent and accurate ab initio parametrization of density functional dispersion correction (DFT-D) for the 94 elements H-Pu, *The Journal of Chemical Physics*, 2010, **132**, 154104.
18. S. Grimme, S. Ehrlich and L. Goerigk, Effect of the damping function in dispersion corrected density functional theory, *Journal of Computational Chemistry*, 2011, **32**, 1456-1465.
19. A. V. Marenich, C. J. Cramer and D. G. Truhlar, Universal Solvation Model Based on Solute Electron Density and on a Continuum Model of the Solvent Defined by the Bulk Dielectric Constant and Atomic Surface Tensions, *The Journal of Physical Chemistry B*, 2009, **113**, 6378-6396.
20. F. Weigend, Accurate Coulomb-fitting basis sets for H to Rn, *Physical Chemistry Chemical Physics*, 2006, **8**, 1057-1065.
